# Iron homeostasis pathway DNA methylation trajectories reveal a role for STEAP3 metalloreductase in patient outcomes after aneurysmal subarachnoid hemorrhage

**DOI:** 10.1186/s43682-021-00003-5

**Published:** 2021-12-20

**Authors:** Lacey W. Heinsberg, Daniel E. Weeks, Sheila A. Alexander, Ryan L. Minster, Paula R. Sherwood, Samuel M. Poloyac, Sandra Deslouches, Elizabeth A. Crago, Yvette P. Conley

**Affiliations:** 1 Department of Human Genetics, Graduate School of Public Health, University of Pittsburgh, Pittsburgh, PA 15261, USA.; 2 Department of Biostatistics, Graduate School of Public Health, University of Pittsburgh, Pittsburgh, PA, USA.; 3 Department of Acute and Tertiary Care, School of Nursing, University of Pittsburgh, Pittsburgh, PA, USA.; 4 College of Pharmacy, University of Texas at Austin, Austin, TX, USA.; 5 Department of Health Promotion and Development, School of Nursing, University of Pittsburgh, Pittsburgh, PA, USA.

**Keywords:** Epigenetics, Biomarker, Group-based trajectory analysis, Six-transmembrane epithelial antigen of prostate 3, Stroke

## Abstract

**Background:**

Following aneurysmal subarachnoid hemorrhage (aSAH), the brain is susceptible to ferroptosis, a type of iron-dependent cell death. Therapeutic intervention targeting the iron homeostasis pathway shows promise for mitigating ferroptosis and improving recovery in animal models, but little work has been conducted in humans. DNA methylation (DNAm) plays a key role in gene expression and brain function, plasticity, and injury recovery, making it a potentially useful biomarker of outcomes or therapeutic target for intervention. Therefore, in this longitudinal, observational study, we examined the relationships between trajectories of DNAm in candidate genes related to iron homeostasis and acute (cerebral vasospasm and delayed cerebral ischemia) and long-term (Glasgow Outcome Scale [GOS, unfavorable = 1–3] and death) patient outcomes after aSAH.

**Results:**

Longitudinal, genome-wide DNAm data were generated from DNA extracted from post-aSAH cerebrospinal fluid (*n* = 260 participants). DNAm trajectories of 637 CpG sites in 36 candidate genes related to iron homeostasis were characterized over 13 days post-aSAH using group-based trajectory analysis, an unsupervised clustering method. Significant associations were identified between inferred DNAm trajectory groups at several CpG sites and acute and long-term outcomes. Among our results, cg25713625 in the STEAP3 metalloreductase gene (*STEAP3*) stood out. Specifically, in comparing the highest cg25713625 DNAm trajectory group with the lowest, we observed significant associations (i.e., based on *p*-values less than an empirical significance threshold) with unfavorable GOS at 3 and 12 months (*OR* = 11.7, *p* = 0.0006 and *OR* = 15.6, *p* = 0.0018, respectively) and death at 3 and 12 months (*OR* = 19.1, *p* = 0.0093 and *OR* = 12.8, *p* = 0.0041, respectively). These results were replicated in an independent sample (*n* = 100 participants) observing significant associations with GOS at 3 and 12 months (*OR* = 8.2, *p* = 0.001 and *OR* = 6.3, *p* = 0.0.0047, respectively) and death at 3 months (*OR* = 2.3, *p* = 0.008) and a suggestive association (i.e., *p*-value < 0.05 not meeting an empirical significance threshold) with death at 12 months (*OR* = 2.0, *p* = 0.0272). In both samples, an additive effect of the DNAm trajectory group was observed as the percentage of participants with unfavorable long-term outcomes increased substantially with higher DNAm trajectory groups.

**Conclusion:**

Our results support a role for DNAm of cg25713625/*STEAP3* in recovery following aSAH. Additional research is needed to further explore the role of DNAm of cg25713625/*STEAP3* as a biomarker of unfavorable outcomes, or therapeutic target to improve outcomes, to translate these findings clinically.

## Background

Aneurysmal subarachnoid hemorrhage (aSAH) is a devastating type of stroke with substantial variability in patient recovery and outcomes. The 30-day fatality rate falls between 25 and 50% [1, 2], and approximately 60% of those who survive experience a range of symptoms and disability that can affect the ability to perform essential functions such as activities of daily living, returning to work, and maintaining healthy relationships [3, 4]. Poor outcomes post-hospitalization have been linked to brain injury that occurs during the first 2 weeks post-aSAH, including acute complications such as cerebral vasospasm (CV) and delayed cerebral ischemia (DCI) [1]. Despite the strong association between CV, DCI, and long-term functional outcomes, the pathophysiology of these complications remains largely unknown and interventions targeting early brain injury have not been successful in improving long-term outcomes post-aSAH [5].

Iron and related homeostatic mechanisms are potentially important factors in response to aSAH [6–11]. Normally, iron is tightly bound to carrier proteins as ferric iron [12]. However, after aSAH, blood contaminates the subarachnoid space and cerebrospinal fluid (CSF) where heme is broken down into carbon monoxide, biliverdin, and non-protein-bound ferrous iron (i.e., free iron) [13]. “Ferroptosis” is a unique type of iron-dependent cell death that results in secondary ischemic brain injury following experimental subarachnoid hemorrhage [14, 15]. Iron chelators effectively reduce associated neuronal cell death in animal models of neurologic injury [15–18], and preliminary research in humans supports the potential importance of iron homeostasis in recovery from aSAH [6, 7, 10, 19].

DNA methylation (DNAm) plays a key role in gene expression and has a significant impact on adult brain function, plasticity, and injury recovery [20], making it a potentially useful biomarker of outcomes or therapeutic target for intervention to improve outcomes after aSAH. While little DNAm research has been performed in the aSAH population specifically, peripheral blood DNA hypomethylation of a candidate region of the genome has been associated with increased mortality after ischemic stroke [21] and global DNA hypomethylation in damaged brain tissue has been observed post-injury in rats after traumatic brain injury [22]. Because DNAm is tissue- and time-sensitive, examining DNAm in CSF acutely post-aSAH may uncover important evidence about the pathophysiology of patient recovery and un-favorable outcomes as the CSF clears, particularly in the context of genes critical to iron homeostasis.

Previously, we developed an objective protocol to largely automate group-based trajectory analysis (GBTA) of longitudinal CSF DNAm data using an exemplar “master” iron regulatory gene, hepcidin (*HAMP*) [9]. GBTA, an unsupervised clustering method, is an ideal approach for clinical examination of DNAm data as it allows one to test if the sample is composed of distinct groups, each with a different underlying trajectory. The identification of DNAm sites that vary over time — and are associated with later outcomes after aSAH — may be useful in identifying subpopulations of patients that require more intensive clinical management to facilitate optimal stroke care delivery and improve patient outcomes. In addition to understanding associations with acute outcomes, it is important to know if CSF DNAm trajectories are associated with long-term outcomes either directly or indirectly via an unmeasured acute complication of vascular or microvascular dysfunction. Therefore, the purpose of this study was to investigate associations between inferred DNAm trajectory groups in candidate genes related to iron homeostasis and acute and long-term patient outcomes following aSAH.

## Results

### Sample characteristics and overview of study workflow and findings

**Fig. 1:**
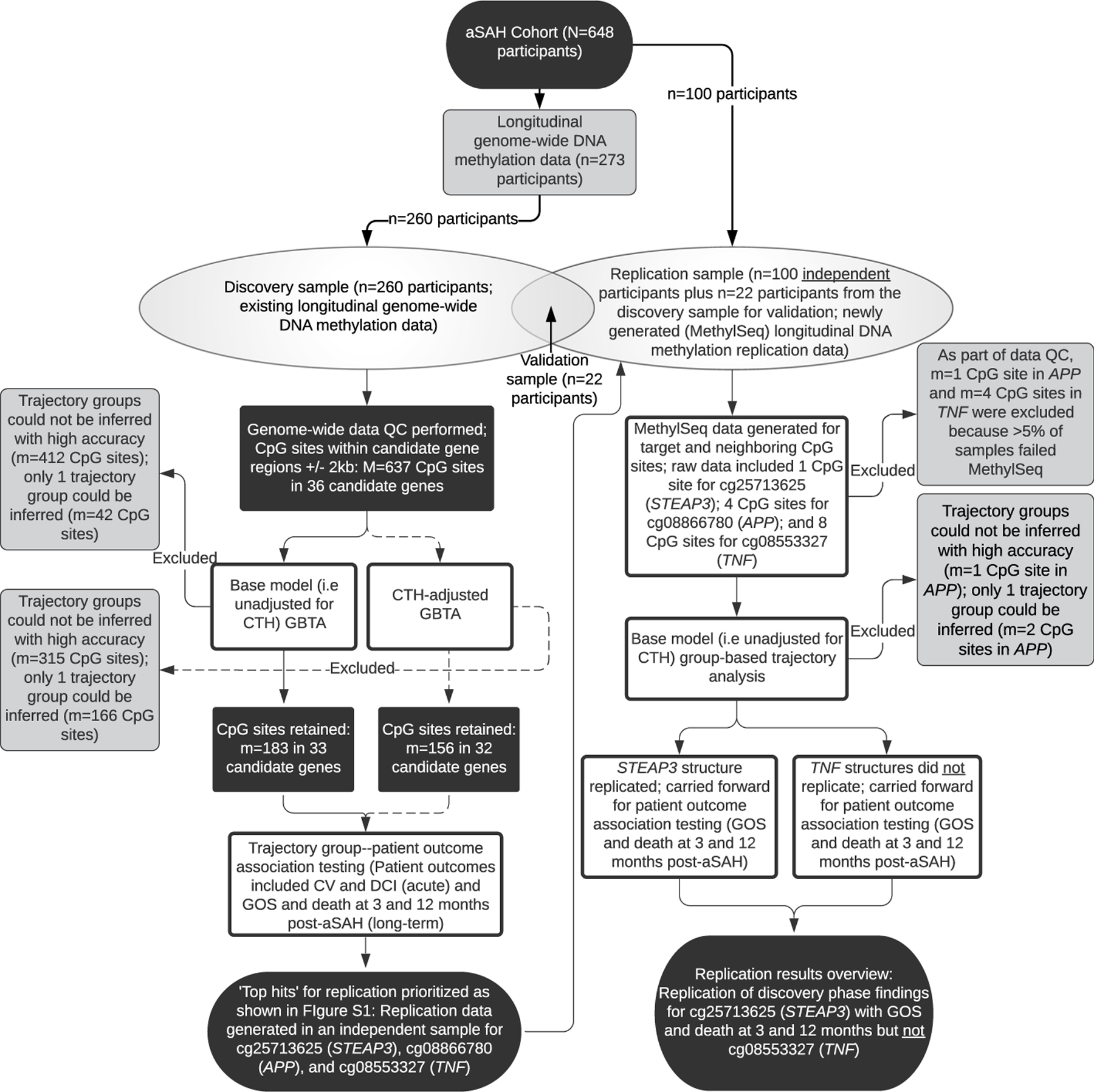
Overview of study workflow and findings. aSAH, aneurysmal subarachnoid hemorrhage; QC, quality control; GBTA, group-based trajectory analysis; CTH, cell type heterogeneity; CV, cerebral vasospasm; DCI, delayed cerebral ischemia; GOS, Glasgow Outcome Scale; *N*/*n* notation refers to the number of participants; *M*/*m* notation refers to the number of CpG sites; dashed lines represent CTH-adjusted analyses, which were considered a secondary focus of this study given our search for a clinical biomarker; candidate genes correspond to those shown in Table S1

As shown in the Fig. 1 study overview and workflow, our final discovery and replication samples consisted of 260 and 100 participants, respectively, with longitudinal DNAm data. Thirty-nine candidate genes were selected based on their known biological roles in iron homeostasis and are listed in the Supplementary Information (SI, Table S1) and described in detail as part of our previous, related work [9, 10].

Sample characteristics are presented in Table 1. Primary acute outcomes of interest included CV and DCI (occurring during the first 2 weeks post-aSAH), and long-term outcomes included Glasgow Outcome Scale (GOS, unfavorable = 1–3) and death at 3 and 12 months post-aSAH. Longitudinal CSF DNAm data were available for participants over 13 and 14 days post-injury in the discovery and replication analyses, respectively, as detailed in the methods. Participants had between 2 and 5 DNAm observations with an average of 3.2 observations in our discovery sample and between 2 and 4 DNAm observations with an average of 3.0 observations in our replication sample. Details of the number of samples available per day are presented in the SI (Table S2 and Fig. S2).

In the entire aSAH cohort (*N* = 648 participants), older age was associated with DCI occurrence (*p* = 0.04), unfavorable GOS at 3 and 12 months (*p* = 0.03 and *p* = 0.04, respectively), and death at 3 and 12 months (*p* = 0.02 and *p* = 0.04). Self-reported race was associated with GOS at 3 months (*p* = 0.04), with individuals who self-identified as White having a higher percentage of favorable GOS scores. Higher Fisher grade was associated with the occurrence of CV (*p* = 0.01) and DCI (*p* = 0.03), as well as unfavorable GOS and death measured at 3 and 12 months (all *p* < 0.001). As such, age, self-reported race, and Fisher grade were included as covariates in our logistic regression models. No significant associations between sex (assigned at birth) and patient outcomes or intervention (surgical vs. coil embolization) and patient outcomes were observed. However, sex was included in our models given the importance of estrogen response elements in iron homeostasis [23].

From the genome-wide data, DNAm data for 36 candidate genes and 637 CpG sites were available and analyzed as part of the targeted discovery phase (Table S1). During data screening, DNAm observations that were identified as extreme outliers > 3 times the interquartile range were score adjusted to prevent bias in GBTA (Table S1) as justified in the SI. As detailed in the methods, to both evaluate the clinical utility of our work and aid in biological interpretation, we performed GBTA both with and without adjusting for cell type heterogeneity (CTH). For our base modeling (i.e., unadjusted for CTH), which was the primary focus of this study given the increased likelihood of clinical utility, our objective protocol eliminated 412 CpG sites as trajectory groups could not be inferred with high accuracy. At an additional 42 CpG sites, we inferred only one trajectory group. After elimination of these sites from our analysis, 183 CpG sites in 33 of our candidate genes were carried forward for patient outcome association testing (Fig. 1). A summary of our CTH-adjusted workflow is provided in parallel (Fig. 1). In examining associations between site-specific inferred DNAm trajectory groups and patient outcomes, we reported unadjusted *p*-values throughout, but judged significance based on empirical thresholds as detailed in the methods. An overview of the results of associations with a *p*-value < 0.05 are presented in the SI for base models (Table S3) and CTH-adjusted models (Table S4) and are detailed in gene-specific tables (Additional File 2).

**Fig. 2:**
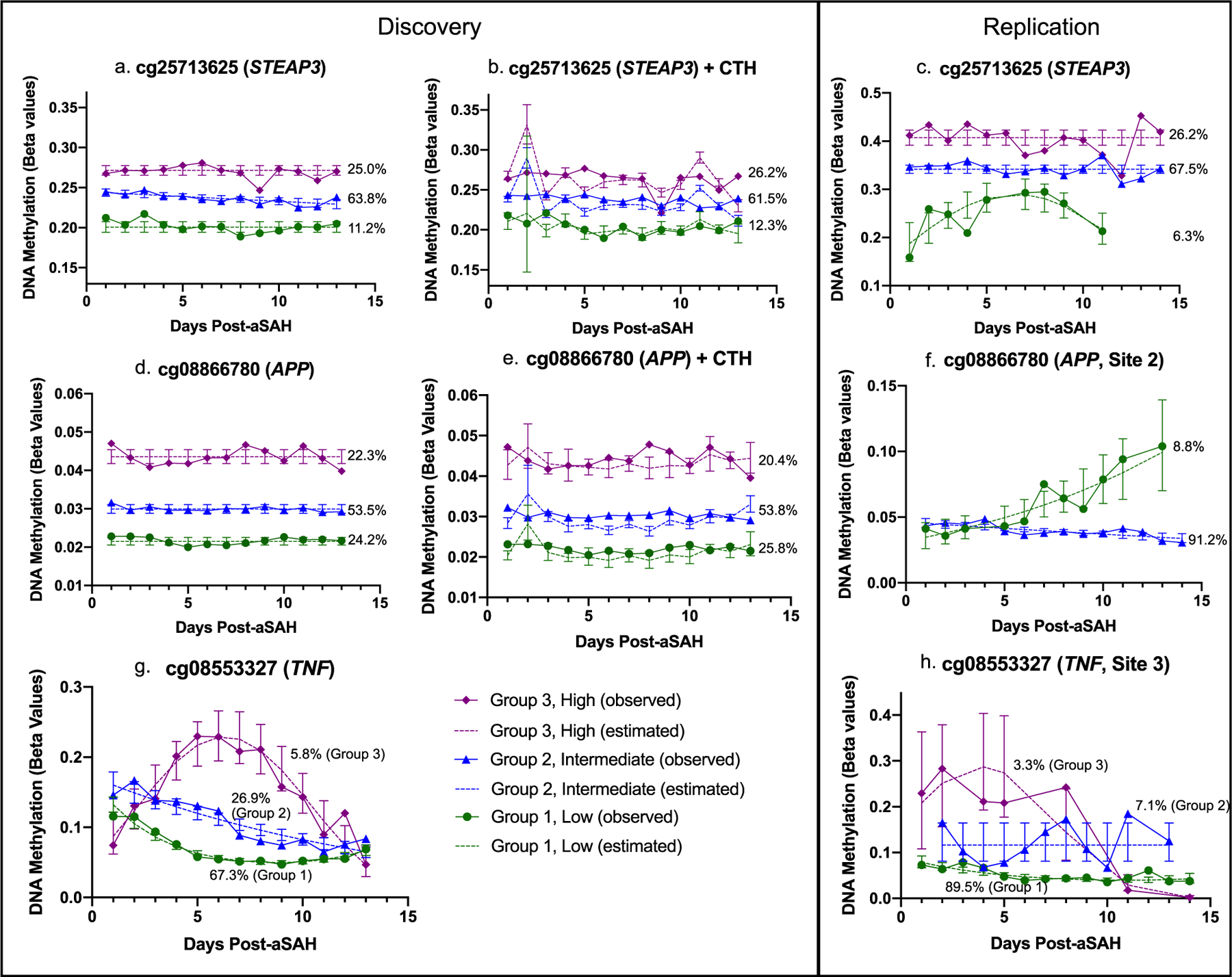
Discovery and replication DNA methylation trajectory plots for top hits. Inferred trajectory groups computed using group-based trajectory analysis separated by discovery phase (base models and CTH-adjusted models) and replication phase (base models only) with group membership percentages depicted; *y*-axis scale, 0 to 1; the CTH-adjusted trajectory model for cg08553327 (*TNF*) did not pass posterior model quality control and therefore is not included here; bars plot the 95% confidence interval for the estimate; DNA methylation data were analyzed as *M* values and converted to beta values for plot presented here; a table with exact DNA methylation levels as measured by beta values is presented in Table S6

Three CpG sites in three candidate genes were flagged as noteworthy of replication as outlined in the SI (Fig. S1 and Table S5). These sites included cg25713625 in STEAP3 metalloreductase (*STEAP3*), cg08866780 in the amyloid precursor protein (*APP*), and cg08553327 in tumor necrosis factor (*TNF*). Given the breadth of this study, we discuss the discovery and replication results for only these three CpG sites, but results of all discovery analyses are presented in the SI as described above. Discovery phase trajectory plots for our top three hits in *STEAP3*, *APP*, and *TNF* are presented in Fig. 2 and in the SI (Fig. S2 and Table S6). Discovery phase participant characteristics by trajectory group are presented in the SI (Tables S7 and S8). It should be noted, however, that the base trajectory models are not directly comparable to the CTH-adjusted trajectory models as group membership changes slightly between the analyses (Fig. S3). Discovery phase binary logistic regression results exploring associations of inferred DNAm trajectory groups with patient outcomes are presented for our top hits in Table 2.

As described below, probe sequences for replication by pyrosequencing (i.e., MethylSeq) were designed to capture top hits and regions surrounding top hits. For cg08866780 (*APP*) and cg08553327 (*TNF*), this captured the target CpG sites and additional variable CpG sites in this region for a total of 4 sites (*APP*) and 8 sites (*TNF*). After replication data quality control (QC), for our top hits of interest in *STEAP3*, *APP*, and *TNF*, we examined a total of 1, 3, and 4 replication sites, respectively.

### Detailed discovery and replication results

#### cg25713625 (STEAP3)

For cg25713625 (*STEAP3*), in the discovery and replication base models (i.e., unadjusted for CTH, Fig. 2a and c, respectively), we inferred three trajectory groups with similar group membership percentages including a low DNAm group (group 1, 11.2% [discovery], 6.3% [replication]), intermediate DNAm group (group 2, 63.8% [discovery], 67.5% [replication]), and high DNAm group (group 3, 25.0% [discovery], 26.2% [replication]). Trajectory shapes appeared similar between the discovery and replication samples, though the low DNAm group had a quadratic shape during replication. After controlling for CTH in the discovery phase (Fig. 2b), we observed nearly identical trajectories and group membership percentages to those observed in the base model. Participant characteristics by base model trajectory groups are presented for the discovery and replication phase in Table 3 and by CTH-adjusted model trajectory groups for the discovery phase in the SI (Tables S7 and S8). As shown in Table 3, in the discovery phase, the low DNAm group had fewer female participants (*p* = 0.011) and individuals who self-reported their race as white (*p* = 0.013) while in the replication phase, the low DNAm group had a higher mean age (*p* = 0.03). No other participant differences by trajectory group were observed.

In the discovery phase association analyses (Table 2a), significant associations (i.e., *p*-value less than the empirical significance threshold computed in permutation testing) were identified with unfavorable GOS at 3 and 12 months (*p* = 0.0006 and *p* = 0.002, respectively) and suggestive associations (i.e., *p*-value < 0.05 not meeting the empirical significance threshold) were identified with death at 3 and 12 months (*p* = 0.009 and *p* = 0.004, respectively). Overall, we observed an increased odds of unfavorable long-term outcomes (*OR* > 11 for all) in the high DNAm group compared with the low DNAm group. In our CTH-adjusted models, suggestive associations were again observed with GOS at 3 and 12 months (*p* = 0.03 and *p* = 0.02, respectively). No significant associations between inferred trajectory groups at cg25713625 and acute outcomes of CV or DCI were observed.

In our replication analyses, a similar additive effect of inferred DNAm trajectory groups was observed as shown in Table 4. As in the discovery phase, the percentage of participants with unfavorable outcomes increased substantially with each inferred DNAm trajectory group. For example, in our replication sample, 0% of participants in the low DNAm trajectory group had unfavorable GOS at 3 months compared with 61.5% of participants in the high DNAm trajectory group.

In the formal replication association analyses presented in Table 5, based on the additive effect of trajectory groups shown in Table 4, the low and intermediate DNAm trajectory groups were combined to stabilize regression parameters (necessary due to the low group’s small sample size). Our discovery phase findings replicated with an increased odds of unfavorable outcomes observed in the high DNAm group compared with the low/intermediate DNAm groups. Specifically, we observed significant associations with unfavorable GOS at 3 and 12 months (*OR* = 8.19, *p* = 0.001 and *OR* = 6.257, *p* = 0.005, respectively) and death at 3 months (*OR* = 2.25, *p* = 0.008). A suggestive association was also observed with death at 12 months (*OR* = 2.03, *p* = 0.027) though it did not reach our empirical significance threshold of 0.0241 to adjust for multiple testing.

### Replication, polynomial order 002, Fig. 2c

#### Group 3 (high) vs. combined group 1 (low) and group 2 (intermediate)

In post hoc cross-sectional analyses, we observed a persistent association between continuous DNAm at the *STEAP3* site (ignoring trajectory groups) and patient outcomes at time 2 (days 3 to 5 post-aSAH), time 3 (days 6 to 8 post-aSAH), and time 4 (days 9 to 11 post-aSAH), with higher DNAm being associated with an increased odds of unfavorable long-term outcomes (Table S9). As described in the Materials and methods section, we also had genome-wide blood DNAm data available in a subset of 67 participants on days 1 to 2 post-aSAH. To understand the potential utility of blood as a surrogate for CSF, we explored the correlation between cg25713625 CSF and blood DNAm data in the subset of participants with both tissues (Table S10) and observed a small to moderate correlation (*R* = 0.36, *p* = 0.0029, Fig. S4). In formal logistic regression analyses, no significant associations between continuous DNAm in neither CSF nor blood were observed on days 1–2 post-aSAH in the small subset of participants with both tissues (Table S11).

#### cg08866780 (APP)

For cg08866780 (*APP*), in the discovery phase base model, we inferred three flat trajectory groups including a low DNAm group (group 1, 24.2%), intermediate DNAm group (group 2, 53.5%), and high DNAm group (group 3, 22.3%) (Fig. 2d). Even after correcting for CTH, we observed very similar trajectory patterns at this CpG site (Fig. 2e). Participant characteristics by trajectory group are presented in the SI (Tables S7 and S8). In discovery phase association analyses, significant associations were identified with favorable GOS at 12 months (*p* = 0.0001) and survival at 3 and 12 months (*p* = 0.001 and *p* = 0.0006, respectively) and a suggestive association was identified with GOS at 3 months (*p* = 0.006). Overall, we observed that participants in the intermediate DNAm group (group 2) had a decreased odds of unfavorable outcomes compared with low and high DNAm groups (groups 1 and 2, respectively) (Table 2b). These results were consistent even after correction for CTH with suggestive associations identified between inferred trajectory groups and favorable GOS at 3 and 12 months (*p* = 0.004 and *p* = 0.01, respectively) and survival at 3 and 12 months (*p* = 0.02 and *p* = 0.01, respectively) (Table 2). No significant associations between inferred trajectory groups at cg08866780 and acute outcomes of CV or DCI were observed.

In our replication analyses, the observed structure did not replicate in any of the three *APP* sites evaluated (Fig. 2f and Fig. S5) nor did the models meet our post-GBTA diagnostic criteria (Table S12). Therefore, no *APP* sites were carried forward for patient outcome association testing in the replication phase.

#### cg08553327 (TNF)

Finally, for cg08553327 (*TNF*), in the discovery base models (i.e., unadjusted for CTH), we inferred three trajectory groups including a low DNAm group (group 1, 67.3%), intermediate DNAm group (group 2, 26.9%), and high DNAm group (group 3, 5.8%). In contrast to the CpG sites discussed above, DNAm at this site was quite dynamic after aSAH (Fig, 2g) and the TNF gene housed a hotspot of several CpG sites that were significantly associated with patient outcomes after aSAH and had similar trajectory shapes as shown in the SI (Table S5 and Additional File 2). CpG site cg08553327 was specifically chosen for replication because it had the smallest *p*-value in the gene and was amenable to MethylSeq. While the CTH-adjusted trajectory model at cg08553327 did not meet our post-GBTA diagnostic criteria, two other sites within the hotspot did and resulted in three steadily decreasing trajectory groups. At these sites, after correction for CTH, suggestive associations persisted between inferred trajectory groups and GOS at 3 months (Additional File 2). Participant characteristics by trajectory group are presented in the SI (Tables S7 and S8). In discovery phase association analyses, significant associations were identified with unfavorable GOS at 3 months (*p* = 0.0003) and suggestive associations were identified with unfavorable GOS at 12 months (*p* = 0.02) and death at 3 and 12 months (*p* = 0.009 and *p* = 0.004, respectively). Overall, we observed that participants in both the intermediate and high DNAm groups had an increased odds of unfavorable outcomes (Table 2c). No significant associations between inferred trajectory groups at cg08553327 and acute outcomes of CV or DCI were observed.

In our replication analyses, the observed structure in the discovery data partially replicated in shape, but not group membership at *TNF* site 3 (Fig. 2h) but not in sites 1, 2, or 4 (Fig. S6). Only the trajectory models for sites 3 and 4 met our post-GBTA diagnostic criteria (Table S12) and were carried forward for patient outcome association testing. None of the observed associations with patient outcomes replicated (Table S13).

## Discussion

Examining DNAm of CSF, a tissue that is proximal to the central nervous system and drained as part of clinical management to reduce intracranial pressure post-aSAH, offered a unique insight into patient recovery, particularly in the context of iron homeostasis post-hemorrhage. Of the candidates examined, associations from three CpG sites in three genes were examined in replication testing including cg25713625 (*STEAP3*), cg08866780 (*APP*), and cg08553327 (*TNF*). Of these, our replication analyses supported nearly identical inferred DNAm trajectory groups and relationships with patient outcomes for cg25713625 (*STEAP3*, UCSC Genome Browser GRCh37/hg 19, chr2: 120022835). Specifically, as the inferred DNAm trajectory group level increased, participants had an increased odds of unfavorable GOS and death at 3 and 12 months.

*STEAP3* was selected as a candidate gene for this study given its primary role in iron transport and homeostasis. Specifically, metalloreductase STEAP3 (Steap3), also known as six-transmembrane epithelial antigen of prostate 3, the protein encoded by the *STEAP3* gene, converts the stable but insoluble ferric form of iron to a less stable but soluble ferrous form [24]. Steap3 deficiency leads to impaired iron homeostasis and presents clinically as microcytic anemia with iron overload [24]. In addition to iron overload as a primary mechanism contributing to ferroptosis, accumulation of lethal, lipid-based reactive oxygen species has also been found to be important [25, 26]. Specifically, elevated free iron post-aSAH has great potential to cause oxidative degradation of lipids that make up cell membranes. For example, in animal models of subarachnoid hemorrhage, elevated iron caused an increase in lipid peroxides and administration of ferrostatin-1, a lipophilic antioxidant which protects cell membranes from lipid oxidation, in turn decreased free iron and improved lipid peroxidation [25]. This intervention successfully prevented both ferroptosis and early brain injury, but had no effect on apoptosis [25]. Beyond this preclinical work, the role of lipid peroxidation metabolites (e.g., 20-HETE) has been associated with unfavorable outcomes after aSAH in humans [27]. Increased levels of Steap3, though essential to normal iron homeostasis, can result in degradation of the cellular membrane through lipid peroxidation, leading to failure of hemolysis and clearance of red blood cells [28], further highlighting the potential importance of this gene.

In addition to iron homeostasis and lipid peroxidation, knockdown of *STEAP3* has been shown to inhibit cell progression suggesting this protein may also play a role in downstream responses to p53, including promoting apoptosis [29]. Steap3 is also thought to be important in immunity as iron deficiency confers resistance to the risk of infection [24]. *STEAP3*, the only STEAP family member to be highly expressed in macrophages, has been shown to be down-regulated by lipopolysaccharide administered as a surrogate for infection in rats [24]. Our CpG site of interest, cg25713625, is annotated to a south shore in the 3′ untranslated region of *STEAP3.* Aside from associations with age in whole blood in children [30–32], there is no significant literature directly related to cg25713625. Interestingly, however, *STEAP3* has been identified as a differentially expressed gene in the intracranial aneurysm wall compared with a control tissue of the superficial temporal artery [33]. Specifically, in a study of 6 participants with unruptured intracranial aneurysms, the authors observed a fold change > 2.5 with higher *STEAP3* expression observed in aneurysm tissue [33].

A surprising finding of this study was that, with the exception of the low DNAm trajectory group in the replication sample, we observed little change over time in the trajectories inferred at this CpG site (Fig. 2a–c). These data suggest that DNAm at this site may not respond dynamically as hypothesized, at least acutely post-aSAH, and that longitudinal data are likely not needed for use as a clinical biomarker. We confirmed this in post hoc cross-sectional analyses in which we observed largely persistent associations between continuous DNAm (ignoring trajectory groups) and long-term outcomes (Table S9). Though these associations were not significant at cross-sectional time 1 (days 1 to 2 post-aSAH) or cross-sectional time 5 (days 12 to 13 post-aSAH), the odds ratios were generally all in the expected direction. Notably, even when correcting for CTH in our discovery analyses, similar associations were observed at this CpG site, suggesting the association is not dependent on changing cell type proportions, further increasing its potential clinical utility.

In comparing cg25713625 DNAm in blood and CSF, we observed only a small to moderate correlation, suggesting blood might not be an appropriate surrogate for this *STEAP3* CSF biomarker (Fig. S4). In our formal regression exploring this further, while there were no significant associations in either tissue, the odds ratios were in the expected direction in CSF (*OR* > 1), but not blood (*OR* < 1), further supporting the idea that blood might not be an appropriate surrogate (Table S11). In any case, these exploratory post hoc analyses were conducted in a small subset of the data (*n* = 67 participants), which greatly limits the interpretability. Studies in larger samples are needed.

We also selected *APP* as a candidate gene for inclusion in this study as amyloid precursor protein is highly expressed in the human brain and ubiquitously expressed across many human tissues [34, 35]. Specific to iron homeostasis, the role of *APP* in managing iron levels is feedback-regulated as iron influx is an important driver in translational expression of neuronal *APP* via an iron-responsive element and, in turn, *APP* plays a role in iron efflux by stabilizing the heavy subunit of ferritin and ferroportin-1 at the binding site [34]. Likewise, *TNF* was selected for inclusion as tumor necrosis factor is a pro-inflammatory cytokine most commonly recognized for its role in inflammation [36]. Iron homeostasis and inflammation are intimately tied as iron deficiency confers resistance to risk of infection and improves inflammatory conditions (e.g., anemia of inflammation in chronic disease). *TNF* plays an important role in iron homeostasis and host defense through regulating inflammation and hypoferremia [37]. Based on our discovery analyses, sites in *APP* (cg08866780, UCSC Genome Browser GRCh37/hg 19, chr21: 27543523) and *TNF* (cg08553327, UCSC Genome Browser GRCh37/hg 19, chr6: 31543647) were selected for replication. We were unable to replicate our findings, however, suggesting DNAm at the two sites examined are not likely important biomarkers or clinical targets post-aSAH.

### Strengths and limitations

There were many strengths to this study. First, the DNAm data were quite unique and available in a relatively large sample of aSAH participants compared with other patient outcome studies post-aSAH. In addition to being longitudinal in nature spanning the acute recovery period post-aSAH, these data were generated from post-aSAH CSF, an understudied tissue. Likewise, we had blood DNAm data in a subset of participants for comparison. Using these data and our objective GBTA approach, we were able to identify dynamic changes in CSF DNAm following aSAH. Furthermore, because genome-wide DNAm data existed in our discovery sample, we were able to examine the effects of CTH. CTH is critical in *biological interpretation* of DNAm studies as differences in cell type proportions within biosamples can impact the overall DNAm level. In the case of post-aSAH CSF, biological interpretation of results presents even greater challenges given the immediate contamination of CSF by peripheral blood, vessel tissue, and brain tissue, but gradual clearing during the acute recovery period. In the case of *clinical interpretation*, however, it matters less if the biomarker association is attributed to DNAm related to changing CTH for that site — or simply the site itself without regard to CTH — as long as a signal can be replicated. While CTH can be controlled for if genome-wide DNAm data are collected [38], it is currently not practical to do so for a clinical biomarker given the associated expense of either cell-sorting or generating genome-wide DNAm data.

Additional strengths of this study included rigorous data QC, imbedded replication, and collection of validation samples (i.e., DNAm data collected for 78 samples from 22 participants using both the discovery and replication platforms). In examining these data, we observed a strong correlation between discovery and replication DNAm data (*R* = 0.74), though the replication data were shifted up by approximately 10% (Fig. S7). Furthermore, in contrast to traditional GBTA, which requires iterative modeling and subjective decision-making from researchers, the GBTA applied here was objective and reproducible as it was largely automated and used a predefined decision-making tree and modeling protocol as detailed else-where [9]. Not only did we replicate both the structure and associations observed for the *STEAP3* site in an independent sample of participants, but we were also able to perform a sensitivity GBTA which included the 22 overlapping validation participants. Of the 22 participants, 16 were assigned to the same group in the replication phase as in the discovery phase. While 8 participants shifted to a new group in the replication (Fig. S8), in all cases, it was an adjacent group (e.g., low to intermediate rather than low to high), further highlighting the replicability of our protocol.

Despite these strengths, there are some important limitations. First, this study was primarily made up of participants who self-reported their race as White. Although the distribution of race observed is largely consistent with Southwestern Pennsylvania demographics, this limits the generalizability of our findings in other races, ethnicities, and ancestries. These factors should be examined in larger and more diverse samples in the future. Next, we used a candidate gene approach which has important weaknesses [39]. However, we addressed the major concerns of candidate gene studies by carefully correcting for multiple testing and replicating our findings in an independent test sample of participants.

While the objective nature of our automated protocol was an important strength of this study, it also eliminated a large portion of CpG sites at which we could not reliably infer trajectory groups. As such, there may be important signals in these data that were not examined in association testing with patient outcomes. Similarly, our tier 1 analyses were limited to existing genome-wide DNAm data. As part of our genome-wide DNAm data QC pipeline described below, CpG sites located on the X chromosome were removed. Therefore, data for two of our candidate genes (*HEPH* and *HJV*) were not available for analysis despite their roles in iron homeostasis. It is possible that variability in these genes is important to outcomes after aSAH. Finally, due to budgetary limitations, we could not collect replication data for all CpG sites that met significance after correction for multiple testing. While we attempted to be as objective as possible in our prioritization of results for replication, true and important signals may exist for other CpG sites. These sites should be examined in the future, particularly those highlighted in Table S5.

## Conclusion

The results of this study support a role for DNAm of cg25713625 (*STEAP3*) in recovery following aSAH. Based on the inferred DNAm trajectories at cg25713625 observed here, we conclude that longitudinal data are not required as CSF DNAm of this site did not appear to change significantly post-aSAH. Additional research, including functional studies, is needed to better understand ferroptosis post-aSAH and further explore the potential role of CSF DNAm of cg25713625/*STEAP3* as a biomarker of unfavorable outcomes, or therapeutic target to improve outcomes, to translate these findings clinically.

## Materials and methods

### Study design, setting, and sample

This was an ancillary, observational, candidate gene association study that capitalized on an existing cohort of aSAH participants with longitudinal phenotype data, biosamples, and genome-wide DNAm data for a subset of participants (Fig. 1) [40]. This study assessed the relationship between inferred DNAm trajectories of iron homeostasis candidate genes and patient outcomes acutely (first 2 weeks post-aSAH) and in the long-term (at 3 and 12 months post-aSAH) using a two-phase design (targeted discovery and replication).

This study adhered to all ethical considerations and was approved by the Institutional Review Board of the University of Pittsburgh. Following informed consent, participants were prospectively recruited from UPMC Presbyterian neurovascular intensive care unit in Pittsburgh, PA, between 2000 and 2017 if they (1) were aged 18 years and older, (2) were newly diagnosed (≤5 days) with aSAH verified with cerebral angiogram, (3) had a Hunt and Hess grade ≥ 2 and/or Fisher grade ≥ 1, (4) were able to read/speak English, and (5) had no history of debilitating neurological disorders. Additional inclusion criteria for this ancillary study included ventriculostomy insertion as part of clinical management to supply CSF samples and availability of serial CSF samples across 14 days post-aSAH.

### DNA methylation data

#### Discovery phase

Data collection The discovery phase capitalized on existing, longitudinal, genome-wide DNAm data collected for 273 participants at up to five time points over 14 days following aSAH as previously described [41] (Fig. 1). DNAm data were generated from DNA extracted from bagged CSF collected as a standard treatment and changed daily for 14 days following aSAH by trained study staff using sterile procedures. The CSF samples were centrifuged, and the cellular pellet and supernatant were stored at −80° until DNA extraction. DNA was extracted from the cellular pellet using the QIAmp Midi kit (catalog #51185) from Qiagen Corp (Qiagen, Valencia, CA, USA), DNA concentration and quality checks were completed, and bisulfite conversion was performed. Genome-wide DNAm data were generated and scanned using the Infinium HumanMethylation450 Beadchip and Illumina iSCAN (Illumina, Incorporated, San Diego, CA, USA) at Johns Hopkins University’s Center for Inherited Disease Research.

As part of our laboratory QC procedures, samples were placed across 11 plates using several strategies to reduce the impact of technical artifacts as described in detail elsewhere [41]. Technical replicates and DNAm control samples were included to assess the reliability of data. Raw genome-wide DNAm data were analyzed using Genome Studio Software (Illumina, Incorporated, San Diego, CA, USA). In parallel, for a subset of 88 participants, genome-wide DNAm data were generated from peripheral blood at a single time point, targeting the early admission period, as detailed elsewhere [41]. In post hoc analyses detailed below, these data were used to compare DNAm in CSF and blood for our top hits, limiting the data to overlapping CSF and blood samples available on days 1–2 post-aSAH for 67 participants.

##### Genome-wide data QC

Genome-wide data cleaning and QC was implemented using R packages minfi [42] and ENmix [43]. This pipeline included removal of poorly performing and outlying samples based on bisulfite control intensities and detection *p*-values and background and dye bias correction to remove non-specific signals from the DNAm data [43, 44]. To further reduce technical variation related to Infinium 450K platform chemistry (i.e., differences in type I and type II probes) and batch effects (i.e., chip, row, and column effects), functional normalization was performed using the “pre-processfunnorm” function from the minfi package [42, 45]. Additional details of our QC pipeline are summarized elsewhere [41]. Of the available sample, observations from days 0 and 14 were removed due to low sample counts, and 13 participants were dropped because they had only one DNAm data observation which precluded trajectory analysis. Our final discovery sample size was 260 participants.

##### Cell type heterogeneity

Under normal conditions, CSF is clear and contains few cells. Following aneurysm rupture, however, CSF is contaminated with both peripheral blood and tissue from the ruptured aneurysm, which gradually clears as the CSF renews during recovery. Because no reference-based methods exist to control for CTH in post-aSAH CSF, we explored this in detail in the discovery phase by generating estimated cell type proportions from the genome-wide DNAm data using Houseman’s reference-free method [46]. This results in estimated proportions of five putative “cell types” for each biosample which were used in our analyses as detailed below.

##### DNAm data extraction

Thirty-nine candidate genes were selected based on their known biological roles in iron homeostasis and described in detail as part of our previous, related work [9, 10]. DNAm data for our candidate genes were extracted from the gene transcript region ±2000 base pairs upstream and downstream. Data for two genes (*HEPH* and *PGRMC1*) were not available in the cleaned genome-wide data and data for the hepcidin gene (*HAMP*) were analyzed in the pilot work to develop the protocols for this study (Table S1) [9].

#### Replication phase

##### Prioritization of top hits for replication

Following gene-specific data screening and analysis described below, top hits for replication were prioritized based on the strength of the identified associations, consistency of results after adjustment for CTH, and presence of hotspots (i.e., multiple CpG sites near each other associated with outcomes) as detailed in the SI (Fig. S1).

##### DNAm data collection and QC

The replication sample was recruited together with the discovery sample as part of a larger parent cohort (Fig. 1). Longitudinal replication DNAm data were generated from CSF for our top hits using pyrosequencing (i.e., MethylSeq) at the Center for Inherited Disease Research. Using 500 ng of DNA, bisulfite conversion was carried out with the Qiagen Epi-Tect Bisulfite kit and PCR reactions were completed using the Qiagen Pyromark PCR Kit following standard protocols and assays designed by and proprietary to Qiagen. Data were generated using Qiagen Pyromark Q48 Autoprep instrument and were called using the Pyromark Q48 Autoprep 2.4.2 software. Probe sequences were designed to capture top hits and regions surrounding top hits. As part of our QC filtering, sites with > 5% of samples failing MethylSeq were excluded. Individual samples with incomplete conversion were not used for sequencing and individual samples that failed the MethylSeq assay were excluded. Additional replication data collection details are provided in the SI (Section II). In total, MethylSeq data were generated for 122 participants, 22 of which overlapped with the discovery sample for validation purposes. The final replication sample size (i.e., independent participants) was 100 participants (Fig. 1).

##### Patient outcomes

Acute patient outcome measures included the occurrence or absence of CV and DCI within a 14-day window following aSAH. CV was defined as ≥25% cerebral vessel narrowing measured by a neurosurgeon via cerebral angiogram obtained as clinically necessary [9], and DCI was defined as the co-occurrence of neurological deterioration (e.g., new and persistent [> 1 h] neurological deficit) and abnormal cerebral blood flow measured using cerebral angiogram or transcranial Doppler (performed twice a day) [9]. Acute outcomes of CV and DCI were treated as binary (occurrence/absence).

Long-term patient outcome measures included the GOS and death at 3 and 12 months following aSAH. These data were collected by trained study staff via in-person or telephone interviews. The GOS is an indication of a participant’s ability to function on a scale of 1 (death) to 5 (good recovery) and has established validity in people with neurological injury [47]. GOS was dichotomized as good (scores of 4 to 5) or unfavorable (scores of 1 to 3). Death data were obtained from the medical record, caregiver/family report, or the Social Security Death Index and treated as binary (yes/no) at the outcome timepoint of interest. In cases where participants were unable to take part in the interview, a caregiver or family member knowledgeable about the participant’s ability to function was interviewed. All study staff involved in outcome data collection were blinded to DNAm status.

Finally, this study capitalized on existing confounding/covariate data extracted from the medical record or collected in surveys as part of a larger research study. These demographic and clinical data included age, sex, race, treatment/intervention, and Fisher grade which provides a measure of the initial extent of aSAH injury based on the amount and distribution of blood observed on a computed tomography scan.

### Statistical analyses

#### Preliminary analyses

All statistical analyses were conducted using R [48] and SAS (SAS Institute Incorporated, Cary, NC, USA). Standard descriptive statistics were computed for all independent, dependent, and potentially confounding/covariate data given the variable’s level of measurement. Preliminary analyses were conducted to identify potential confounders/covariates. DNAm data were analyzed as *M* values and any value labeled as an extreme outlier (a DNAm value above or below three times the inter-quartile range) was replaced with the maximum or minimum observed DNAm value below the extreme outlier threshold for values on each day. Missing data were assessed and filled in from the medical record when possible. An expanded description of our statistical analyses is provided in the SI (Section III).

#### Group-based trajectory analyses

GBTA, an unsupervised clustering method used to identify clusters of individuals who have similar development trajectories (e.g., DNA methylation trajectory groups post-aSAH), was conducted using Proc TRAJ and a censored normal model in SAS. While other methods of longitudinal modeling (e.g., latent curve analysis) estimate the sample average trajectory and use covariates to explain variability about this average, GBTA is dissimilar in that it assumes the sample is composed of distinct groups, each with a different underlying trajectory. GBTA is a highly flexible approach, allowing for a variety of complexities including missing or sparse data, time-varying covariates, and small sample sizes as it uses the maximum likelihood method to estimate parameters, including group sizes and shapes of trajectories [49, 50]. Therefore, GBTA is an ideal approach to apply in cases where individual observations are available from different time points (e.g., different days post-aSAH) as it generates asymptotically unbiased parameter estimates.

In GBTA, models with a varying number of groups and polynomial orders (i.e., group trajectory shapes) are compared to find the model that best fits the longitudinal data [51]. As part of this process, a trajectory group-specific posterior probability of assignment is computed for each participant. Ultimately, individuals are assigned to the group for which their posterior probability is the highest [51]. Given the subjectivity and iterative modeling required in traditional GBTA, and the large number of candidate genes and CpG sites examined here, the model selection process was largely automated. Specifically, we determined the “best fitting” of 39 possible models with a maximum of three groups and comprehensive combinations of polynomial orders of 0 (intercept only), 1 (linear), and 2 (quadratic). Our GBTA automated protocol has been described in detail as part of our pilot work [9] and summarized in the SI (Section II).

Following GBTA for each CpG site, we performed a secondary evaluation of model adequacy (i.e., evaluation of post-GBTA diagnostics) using several traditional measures including (1) an average posterior probability (AvePP) > 0.7, (2) odds of correct classification (OCC) > 5, (3) estimated group membership (π) > 5%, (4) reasonably close estimated group membership (π) versus the assigned group proportion (P*), and (5) a relatively narrow 95% confidence interval for the estimated group probability (π) [9, 49]. If the trajectory model for an individual CpG site failed for the top two “best” models, we concluded that DNAm trajectory groups could not be inferred with high accuracy for that site and it was excluded from further analysis [9].

To examine the potential clinical utility of DNAm trajectories unadjusted for CTH, as well as to support the biological interpretation of results evaluating potential confounding by cell type, we implemented our GBTA protocol twice for all CpG sites to compute DNAm trajectories both unadjusted for CTH (i.e., base models) and adjusted for CTH [9]. CTH data were generated in our QC pipeline as described above and were controlled for as time-varying covariates during GBTA in our CTH-adjusted models, embedding the additional variables within the trajectory group assignment. GBTA was performed using *M* values and final models and estimates were converted to beta values for presentations in trajectory plots.

##### Patient outcome association testing

Binary logistic regression was performed in R to determine the relationship between inferred trajectory groups for each CpG site and patient outcomes while controlling for age, sex, race, and Fisher grade (i.e., Outcome ~ Trajectory group + age + sex + race + Fisher grade). A partial *F* test was used to produce a global *p*-value of the overall model fit by comparing the full model (including the trajectory group) with a restricted model (omitting the trajectory group). As detailed in the SI (Section III), permutation testing was used to correct for multiple testing of correlated outcomes [9]. Associations with a *p-*value < 0.05 were considered suggestive, and associations with a *p*-value falling below the empirical significance threshold were considered significant.

#### Post hoc analyses

Longitudinal DNAm data were collapsed into cross-sectional time points including time 1 (days 1–2 post-aSAH), time 2 (days 3 to 5 post-aSAH), time 3 (days 6 to 8 post-aSAH), time 4 (days 9 to 11 post-aSAH), and time 5 (days 12 to 14 post-aSAH). Binary logistic regression was performed to determine the relationship between continuous DNAm of top hits (i.e., ignoring trajectory groups) and patient outcomes while controlling for age, sex, race, and Fisher grade within cross-sectional time points. For the subset of participants with both CSF and blood DNAm data on days 0–2 post-aSAH (*n* = 67 participants), *M* values and beta values were compared using scatterplots and Pearson correlation coefficients, and regression lines were drawn to better understand this relationship relative to *y = x.* Finally, binary logistic regression was again performed to explore the relationship between continuous DNAm of top hits in CSF and blood and patient outcomes while controlling for age, sex, race, and Fisher grade.

## Supplementary Material

Supplement1 **Additional file 1:** Section I. Expanded results; **Table S1.** Iron homeostasis gene, data extraction, and extreme outlier summary; **Table S2.** Number of DNA methylation observations by day in discovery and replication samples; **Table S3.** Summary of significant and suggestive associations for base models (no adjustment for CTH); **Table S4.** Summary of significant and suggestive associations for CTH-adjusted models; **Figure S1.** Flow chart for prioritization of findings for replication; **Table S5.** Summary of prioritization of top hits in base models (i.e., unadjusted for CTH to allow for replication by MethySeq); **Figure S2.** Discovery phase spaghetti plots depicting DNA methylation over 13 days post-aSAH for top hits in STEAP3, APP, and TNF; **Table S6.** Discovery and replication levels of DNA methylation as measured by beta values (corresponds with Figure 2); **Table S7.** Discovery phase participant characteristics by trajectory group for cg25713625 (STEAP3), cg08866780 (APP), and cg08553327 (TNF); **Table S8.** Discovery phase patient outcome distributions by base model trajectory groups for cg25713625 (STEAP3), cg08866780 (APP), and cg08553327 (TNF); **Figure S3.** Sankey plots depicting shifts in trajectory group assignment between discovery phase base and CTH-adjusted models; **Table S9.** Results of discovery phase binary logistic regression examining associations of continuous cg25713625 (STEAP3) CSF DNA methylation with patient outcomes while controlling for age, sex, self-identified race, and Fisher grade in cross-sectional time points; **Table S10.** Characteristics of the subset of participants with blood DNA methylation available on days 0–2 post-aSAH; **Figure S4.** Correlation between cg25713625 (STEAP3) CSF and Blood DNA Methylation, Days 0–2 post-aSAH; **Table S11.** Results of binary logistic regression examining associations of continuous cg25713625 (STEAP3) DNA methylation with patient outcomes while controlling for age, sex, self-identified race, and Fisher grade in the subset of participants with both blood and CSF available on days 0–2 post-aSAH in the discovery sample; **Figure S5.** Replication DNA methylation trajectory plots for CpGs near cg08866780 (APP); **Figure S6.** Replication DNA methylation trajectory plots for CpGs near cg08553327 (TNF); **Table S12.** Post-GBTA diagnostic summary for replication data group-based trajectory analysis for sites in STEAP3, APP, and TNF; **Table S13.** Replication results of binary logistic regression examining associations of cg08553327 (TNF), sites 3 and 4, with patient outcomes while controlling for age, sex, self-identified race, and Fisher grade; **Figure S7.** Comparison of cg25713625 (STEAP3) DNA methylation data from validation samples overlapping between discovery and replication data; **Figure S8.** Sankey plot depicting shifts in trajectory group assignment between discovery and replication analyses for cg25713625 (STEAP3) replication; Section II. Expanded replication data collection methods; **Table S14.** Replication assay information for top hits; **Table S15.** Summary of replication data QC (pass, check, fail); **Section III.** Expanded statistical analysis.

Supplement2 **Additional file 2:.** Gene-specific detailed results.

## Figures and Tables

**Table 1:** Participant characteristics for the entire aneurysmal subarachnoid hemorrhage cohort and discovery and replication sample subsets

Variable	aSAH cohort (*n* = 648)		Discovery sample (*n* = 260)		Replication sample (*n* = 100)	
Age, mean years (*SD*)	53.2 (11.5)		53.1 (11.0)		54.2 (12.0)	
Sex, female, *n* (%)	469 (72.4)		179 (68.8)		76 (76.0)	
Self-identified race, white, *n* (%)	563 (86.9)		225 (86.5)		91 (91.0)	
Treatment, coil embolization, *n* (%)	402 (62.0)		159 (61.2)		66 (66.0)	
Fisher grade, *n* (%)						
2	266 (41.0)		78 (30.0)		41 (41.0)	
3	279 (43.1)		126 (48.5)		43 (43.0)	
4	103 (15.9)		56 (21.5)		16 (16.0)	
Outcome	*N*	Unfavorable, *n* (%)	*N*	Unfavorable, *n* (%)	*N*	Unfavorable, *n* (%)
CV	396	206 (52.0)	168	91 (54.2)	NA^a^	
DCI	631	253 (40.1)	258	127 (49.2)		
GOS-3	555	149 (26.8)	214	71 (33.2)	99	38 (38.4)
GOS-12	530	121 (22.8)	204	53 (26.0)	93	29 (31.2)
Death-3	594	88 (14.8)	232	39 (16.8)	99	24 (24.2)
Death-12	530	96 (18.1)	204	44 (21.6)	93	26 (28.0)

*aSAH*, aneurysmal subarachnoid hemorrhage; *SD*, standard deviation; *CV*, cerebral vasospasm (unfavorable = CV present); *DCI*, delayed cerebral ischemia (unfavorable = DCI present); *GOS-3*, Glasgow Outcome Scale at 3 months (unfavorable = 1–3); *GOS-12*, Glasgow Outcome Scale at 12 months (unfavorable = 1–3); *Death-3*, death at 3 months (unfavorable = yes); *Death-12*, death at 12 months (unfavorable = yes);

a CV and DCI were not examined in the replication sample given null association in discovery analyses

**Table 2:** Discovery results of binary logistic regression and global analysis examining associations of cg25713625 (*STEAP3*), cg08866780 (*APP*), and cg08553327 (*TNF*) unadjusted and CTH-adjusted trajectory groups with patient outcomes while controlling for age, sex, self-identified race, and Fisher grade

Table 2a. cg25713625 (*STEAP3*)							
Base model, polynomial order 010, Fig. 2a							
	Group 2 (intermediate) vs. group 1 (low)			Group 3 (high) vs. group 1 (low)			
Outcome	*OR*	*95% CI*	*p* ^c^	*OR*	*95% CI*	*p* ^c^	Global *p*^d^
CV	0.662	0.21 to 2.03	0.471	1.126	0.33 to 3.80	0.847	0.351
DCI	1.032	0.45 to 2.41	0.941	1.256	0.49 to 3.20	0.629	0.789
GOS-3	2.982	0.86 to 12.56	0.1	11.743	3.14 to 21.65	**0.0006** ^a^	**0.00005** ^a^
GOS-12	6.346	1.47 to 31.32	0.029 ^b^	15.595	3.35 to 22.54	**0.0018** ^a^	**0.0005** ^a^
Death-3	7.147	1.00 to 32.53	0.076	19.081	3.01 to 25.12	0.0093 ^b^	**0.0013** ^a^
Death-12	4.859	1.00 to 35.80	0.064	12.762	2.71 to 25.13	0.0041 ^b^	**0.0015** ^a^
CTH-adjusted model, polynomial order 000, Fig. 2b							
	Group 2 (intermediate) vs. group 1 (low)			Group 3 (high) vs. group 1 (low)			
Outcome	*OR*	*95% CI*	*p* ^c^	*OR*	*95% CI*	*p* ^c^	Global *p*^d^
CV	0.433	0.14 to 1.26	0.131	0.832	0.25 to 2.70	0.76	0.129
DCI	0.612	0.27 to 1.36	0.231	0.913	0.37 to 2.20	0.84	0.260
GOS-3	2.589	0.80 to 9.35	0.115	4.143	1.25 to 5.01	0.0271 ^b^	0.062
GOS-12	3.960	1.00 to 10.58	0.055	5.972	1.52 to 6.25	0.0182 ^b^	0.0326 ^b^
Death-3	3.768	0.90 to 11.54	0.108	4.847	1.05 to 7.50	0.069	0.122
Death-12	3.120	0.86 to 15.63	0.115	4.249	1.06 to 5.25	0.059	0.123
**Table 2b. cg08866780 (*APP*)**							
Base model, polynomial order 000, Fig. 2d							
	Group 2 (intermediate) vs. group 1 (low)			Group 3 (high) vs. group 1 (low)			
Outcome	*OR*	*95% CI*	*p* ^c^	*OR*	*95% CI*	*p* ^c^	Global *p*^d^
CV	0.443	0.19 to 0.989	0.051	0.366	0.14 to 1.00	0.056	0.069
DCI	0.803	0.44 to 1.48	0.479	1.156	0.56 to 2.41	0.698	0.488
GOS-3	0.349	0.16 to 0.74	0.0060 ^b^	0.760	0.32 to 1.82	0.541	0.0118 ^b^
GOS-12	0.194	0.08 to 0.44	**0.0001** ^a^	0.826	0.39 to 1.98	0.672	**0.0001** ^a^
Death-3	0.209	0.08 to 0.52	**0.0010** ^a^	1.080	0.44 to 2.67	0.868	**0.0002** ^a^
Death-12	0.206	0.08 to 0.50	**0.0006** ^a^	0.977	0.39 to 2.43	0.96	**0.0002** ^a^
CTH-adjusted model, polynomial order 000, Fig. 2e							
	Group 2 (intermediate) vs. group 1 (low)			Group 3 (high) vs. group 1 (low)			
Outcome	*OR*	*95% CI*	*p* ^c^	*OR*	*95% CI*	*p* ^c^	Global *p*^d^
CV	0.813	0.37 to 1.76	0.602	0.654	0.25 to 1.67	0.374	0.672
DCI	1.230	0.68 to 2.24	0.494	1.400	0.67 to 2.97	0.377	0.656
GOS-3	0.332	0.16 to 0.70	0.0040 ^b^	0.683	0.27 to 1.67	0.4072	0.0097 ^b^
GOS-12	0.347	0.15 to 0.77	0.0097 ^b^	1.070	0.43 to 2.68	0.8853	0.0066 ^b^
Death-3	0.359	0.15 to 0.87	0.0237 ^b^	1.284	0.50 to 3.31	0.6028	0.0091 ^b^
Death-12	0.337	0.14 to 0.79	0.0128 ^b^	1.143	0.44 to 2.95	0.7817	0.0072 ^b^
**Table 2c. cg08553327 (*TNF*)**							
Base model, polynomial order 212, Fig. 2g							
	Group 2 (intermediate) vs. group 1 (low)			Group 3 (high) vs. group 1 (low)			
Outcome	*OR*	*95% CI*	*p* ^c^	*OR*	*95% CI*	*p* ^c^	Global *p*^d^
CV	0.850	0.42 to 1.72	0.651	1.223	0.31 to 5.36	0.777	0.844
DCI	0.827	0.47 to 1.46	0.516	3.066	0.98 to 11.64	0.069	0.101
GOS-3	3.686	1.78 to 7.87	**0.0005** ^a^	13.134	3.51 to 39.02	**0.0003** ^a^	**0.00001** ^a^
GOS-12	2.080	0.98 to 4.43	0.0568	4.488	1.24 to 16.50	0.0209 ^b^	0.0244 ^b^
Death-3	2.488	1.11 to 5.57	0.0257 ^b^	5.603	1.47 to 20.44	0.0089 ^b^	0.0094 ^b^
Death-12	2.496	1.12 to 5.56	0.0243 ^b^	7.123	1.89 to 27.94	0.0037 ^b^	0.0038 ^b^

Corresponds to trajectory groups depictured in Fig. 2a (*STEAP3*), 2b (*STEAP3* + CTH), 2d (*APP*), 2e (*APP* + CTH), and 2g (*TNF*). Base model, unadjusted for CTH; *OR*, odds ratio; *CI*, confidence interval; *p*, *p*-value based on an alpha of 0.05; *Global p*, *p*-value computed using partial *F* test comparing models with and without trajectory group; *CTH*, cell type heterogeneity; *CV*, cerebral vasospasm; *DCI*, delayed cerebral ischemia; *GOS-3*, Glasgow Outcome Scale at 3 months (unfavorable = 1–3); *GOS-12*, Glasgow Outcome Scale at 12 months (unfavorable = 1–3); *Death-3*, death at 3 months; *Death-12*, death at 12 months.

a Significant associations (meeting empirical significance level) have been bolded.

b Suggestive associations (unadjusted *p* < 0.05) have been underlined.

c Empirical significance threshold = 0.002 (calculated based on the minimum of 12 *p*-values, including both group 2 vs. group 1 and group 3 vs. group 1 comparisons, in permutation testing).

d Empirical significance threshold for global analysis = 0.003 (calculated based on the minimum of 6 *p*-values in permutation testing)

**Table 3:** Participant characteristics by trajectory groups for cg25713625 (*STEAP3*) discovery and replication samples

	Discovery (*n* = 260)				Replication (*n* = 100)			
Variable	Group 1 (low)	Group 2 (intermediate)	Group 3 (high)	*p*	Group 1 (low)	Group 2 (intermediate)	Group 3 (high)	*p*
Group membership, *n* (%)	29 (11.1)	166 (63.8)	65 (25.0)		6 (6.0)	68 (68.0)	26 (26.0)	
Age, mean (*SD*)	52.3 (12.2)	53.0 (10.8)	53.6 (11.2)	0.862^a^	65.2 (6.6)	54.4 (12.0)	51.0 (11.6)	**0.03** ^a^
Sex, female, *n* (%)	13 (44.8)	118 (71.1)	48 (73.8)	**0.011** ^b^	4 (66.7)	53 (77.9)	19 (73.1)	0.667^c^
Self-identified race, white, *n* (%)	20 (69.0)	147 (88.6)	58 (89.2)	**0.013** ^b^	6 (100)	61 (89.7)	24 (92.3)	1c
Fisher grade								
*2*	6 (20.7)	54 (32.5)	18 (27.7)	0.558^b^	5 (83.3)	27 (39.7)	9 (34.6)	0.719^b,d^
*3*	14 (48.3)	80 (48.2)	32 (49.2)		1 (16.7)	30 (44.1)	12 (46.2)	
*4*	9 (31.0)	32 (19.3)	15 (23.1)		0	11 (16.2)	5 (19.2)	
Intervention, coil, *n* (%)	16 (55.2)	99 (59.6)	40 (61.5)	0.845^b^	4 (66.7)	46 (67.6)	16 (61.5)	0.875^c^

*Discovery*, trajectory groups depicted in Fig. 2a; *Replication*, trajectory groups depicted in Fig. 2c; *SD*, standard deviation; significant associations based on *p* < 0.05 have been bolded.

a One-way analysis of variance;

b chi-square test of independence;

c Fisher’s exact test;

d computed by combining low and intermediate groups because of 0 cell count

**Table 4:** Outcome distributions by trajectory group in cg25713625 (*STEAP3*) discovery and replication samples

GOS-3						
	Discovery (*n* = 214)			Replication (*n* = 99)		

Group	Favorable, *n*	Unfavorable, *n*	% Unfavorable	Favorable, *n*	Unfavorable, *n*	% Unfavorable
3 (high)	23	29	55.77	10	16	61.54
2 (intermediate)	101	38	27.34	45	22	32.84
1 (low)	19	4	17.39	6	0	0.00
GOS-12						
	Discovery (*n* = 204)			Replication (*n* = 93)		
Group	Favorable, *n*	Unfavorable, *n*	% Unfavorable	Favorable, *n*	Unfavorable, *n*	% Unfavorable
3 (high)	32	22	40.74	12	13	52.00
2 (intermediate)	97	29	23.02	46	16	25.81
1 (low)	22	2	8.33	6	0	0.00
Death-3						
	Discovery (*n* = 232)			Replication (*n* = 99)		
Group	No, *n*	Yes, *n*	% Yes	No, *n*	Yes, *n*	% Yes
3 (high)	40	17	29.82	15	11	42.31
2 (intermediate)	130	21	13.91	54	13	19.40
1 (low)	23	1	4.17	6	0	0.00
Death-12						
	Discovery (*n* = 204)			Replication (*n* = 93)		
Group	No, *n*	Yes, *n*	% Yes	No, *n*	Yes, *n*	% Yes
3 (high)	35	19	35.19	14	11	44.00
2 (intermediate)	103	23	18.25	47	15	24.19
1 (low)	22	2	8.33	6	0	0.00

*Discovery*, trajectory groups depicted in Fig. 2a; *Replication*, trajectory groups depicted in Fig. 2c; *GOS-3*, Glasgow Outcome Scale at 3 months (favorable = 4–5, unfavorable=1–3); *GOS-12*, Glasgow Outcome Scale at 12 months (favorable = 4–5, unfavorable = 1–3); *Death-3*, death at 3 months; *Death-12*, death at 12 months

**Table 5:** Replication results of binary logistic regression examining associations of cg25713625 (*STEAP3*) with patient outcomes while controlling for age, sex, self-identified race, and Fisher grade

Replication, polynomial order 002, Fig. 2c			
Group 3 (high) vs. combined group 1 (low) and group 2 (intermediate)			
Outcome	*OR*	*95% CI*	*p* ^c^
GOS-3	8.190	2.51 to 31.85	**0.0010** ^a^
GOS-12	6.257	1.85 to 24.53	**0.0047** ^a^
Death-3	2.253	1.26 to 4.23	**0.0080** ^a^
Death-12	2.028	1.10 to 3.92	0.0272 ^b^

Corresponds to trajectory groups depicted in Fig. 2c; *GOS-3*, Glasgow Outcome Scale at 3 months (unfavorable = 1–3); *GOS-12*, Glasgow Outcome Scale at 12 months (unfavorable = 1–3); *Death-3*, death at 3 months; *Death-12*, death at 12 months.

a Significant associations (meeting empirical significance level) have been bolded.

b Suggestive associations (unadjusted *p* < 0.05) have been underlined.

c Empirical significance threshold = 0.0241 (calculated based on the minimum of 4 *p*-values in permutation testing)

## Abbreviations

aSAH: Aneurysmal subarachnoid hemorrhage
DNAm: DNA methylation
CSF: Cerebrospinal fluid
CTH: Cell type heterogeneity
CV: Cerebral vasospasm
DCI: Delayed cerebral ischemia
GBTA: Group-based trajectory analysis
SI: Supplementary information
STEAP3: STEAP3 metalloreductase
APP: Amyloid precursor protein
TNF: Tumor necrosis factor
OR: Odds ratio
QC: Quality control
OCC: Odds of correct classification
AvePP: Average posterior probability
