# Supplementary material for: Iron homeostasis pathway DNA methylation trajectories reveal a role for STEAP3 metalloreductase in patient outcomes after aneurysmal subarachnoid hemorrhage
Source: Epigenetics Commun. Author manuscript; Available in PMC 2022 Jan 25. (PMC8788201; doi:10.1186/s43682-021-00003-5)
Supplement: Supplement1 — Additional file 1: Section I. Expanded results; Table S1. Iron homeostasis gene, data extraction, and extreme outlier summary; Table S2. Number of DNA methylation observations by day in discovery and replication samples; Table S3. Summary of significant and suggestive associations for base models (no adjustment for CTH); Table S4. Summary of significant and suggestive associations for CTH-adjusted models; Figure S1. Flow chart for prioritization of findings for replication; Table S5. Summary of prioritization of top hits in base models (i.e., unadjusted for CTH to allow for replication by MethySeq); Figure S2. Discovery phase spaghetti plots depicting DNA methylation over 13 days post-aSAH for top hits in STEAP3, APP, and TNF; Table S6. Discovery and replication levels of DNA methylation as measured by beta values (corresponds with Figure 2); Table S7. Discovery phase participant characteristics by trajectory group for cg25713625 (STEAP3), cg08866780 (APP), and cg08553327 (TNF); Table S8. Discovery phase patient outcome distributions by base model trajectory groups for cg25713625 (STEAP3), cg08866780 (APP), and cg08553327 (TNF); Figure S3. Sankey plots depicting shifts in trajectory group assignment between discovery phase base and CTH-adjusted models; Table S9. Results of discovery phase binary logistic regression examining associations of continuous cg25713625 (STEAP3) CSF DNA methylation with patient outcomes while controlling for age, sex, self-identified race, and Fisher grade in cross-sectional time points; Table S10. Characteristics of the subset of participants with blood DNA methylation available on days 0–2 post-aSAH; Figure S4. Correlation between cg25713625 (STEAP3) CSF and Blood DNA Methylation, Days 0–2 post-aSAH; Table S11. Results of binary logistic regression examining associations of continuous cg25713625 (STEAP3) DNA methylation with patient outcomes while controlling for age, sex, self-identified race, and Fisher grade in the subset of participan [file NIHMS1770578-supplement-Supplement1.docx]

Iron Homeostasis Pathway DNA Methylation Trajectories Reveal a Role for STEAP3 Metalloreductase

in Patient Outcomes after Aneurysmal Subarachnoid Hemorrhage

**Additional File 1: Supplementary Methods and Results**

Lacey W. Heinsberg, PhD, RN^*^; Daniel E. Weeks, PhD; Sheila A. Alexander, PhD, RN, FCCM;

Ryan L. Minster, PhD, MSIS; Paula R. Sherwood, PhD, RN, CNRN, FAAN; Samuel M. Poloyac, PharmD, PhD;

Sandra Deslouches, BS; Elizabeth A. Crago, PhD, RN; Yvette P. Conley, PhD, FAAN

*Corresponding author, e-mail: law145@pitt.edu

**TABLE OF CONTENTS**

| **Section** | **Table/Figure** | **Title** | **Page** |
| --- | --- | --- | --- |
| [*Section I*](#SectionI) |  | Expanded results | 2 |
|  | [Table S1](#Table1) | Iron homeostasis gene, data extraction, and extreme outlier summary | 2 |
|  | [Table S2](#TableS2) | Number of DNA methylation observations by day in discovery and replication samples | 4 |
|  | [Table S3](#TableS3) | Summary of significant and suggestive associations for base models (no adjustment for CTH) | 5 |
|  | [Table S4](#TableS4) | Summary of significant and suggestive associations for CTH-adjusted models | 10 |
|  | [Figure S1](#FigureS1) | Flow Chart for Prioritization of Findings for Replication | 13 |
|  | [Table S5](#TableS5) | Summary of prioritization of top hits in base models (i.e., unadjusted for CTH to allow for replication by pyrosequencing) | 14 |
|  | [Figure S2](#FigureS2) | Discovery phase spaghetti plots depicting DNA methylation over 13 days post-aSAH for top hits in *STEAP3*, *APP*, and *TNF* | 15 |
|  | [Table S6](#TableS6) | Discovery and replication levels of DNA methylation as measured by beta values (corresponds with Figure 2) | 16 |
|  | [Table S7](#TableS7) | Discovery phase participant characteristics by trajectory group for cg25713625 (*STEAP3*), cg08866780 (*APP*), and cg08553327 (*TNF*) | 17 |
|  | [Table S8](#TableS8) | Discovery phase patient outcome distributions by base model trajectory groups for cg25713625 (*STEAP3*), cg08866780 (*APP*), and cg08553327 (*TNF*) | 18 |
|  | [Figure S3](#FigureS3) | Sankey plots depicting shifts in trajectory group assignment between discovery phase base and CTH-adjusted models | 19 |
|  | [Table S9](#TableS9) | Results of discovery phase binary logistic regression examining associations of continuous cg25713625 (*STEAP3*) CSF DNA methylation with patient outcomes while controlling for age, sex, self-identified race, and Fisher grade in cross-sectional time points | 20 |
|  | [Table S10](#TableS10) | Characteristics of the subset of participants with blood DNA methylation available on days 1-2 post-aSAH | 21 |
|  | [Figure S4](#FigureS4) | Correlation between cg25713625 (*STEAP3*) CSF and Blood DNA Methylation, Days 1-2 post-aSAH | 22 |
|  | [Table S11](#TableS11) | Results of binary logistic regression examining associations of continuous cg25713625 (*STEAP3*) DNA methylation with patient outcomes while controlling for age, sex, self-identified race, and Fisher grade in the subset of participants with both blood and CSF available on days 1-2 post-aSAH in the discovery sample | 23 |
|  | [Figure S5](#FigureS5) | Replication DNA methylation trajectory plots for CpGs near cg08866780 (*APP*) | 24 |
|  | [Figure S6](#FigureS6) | Replication DNA methylation trajectory plots for CpGs near cg08553327 (*TNF*) | 25 |
|  | [Table S12](#TableS12) | Post-GBTA diagnostic summary for replication data group-based trajectory analysis for sites in *STEAP3*, *APP*, and *TNF* | 26 |
|  | [Table S13](#TableS13) | Replication results of binary logistic regression examining associations of cg08553327 (*TNF*), sites 3 and 4, with patient outcomes while controlling for age, sex, self-identified race, and Fisher grade | 27 |
|  | [Figure S7](#FigureS7) | Comparison of cg25713625 (*STEAP3*) DNA methylation data from validation samples overlapping between discovery and replication data | 28 |
|  | [Figure S8](#FigureS8) | Sankey plot depicting shifts in trajectory group assignment between discovery and replication analyses for cg25713625 (*STEAP3*) replication | 29 |
| [*Section II*](#SectionII) |  | Expanded replication data collection methods | 30 |
|  | [Table S14](#TableS14) | Replication assay information for top hits | 30 |
|  | [Table S15](#TableS15) | Summary of replication data QC (pass, check, and fail) | 31 |
| [*Section III*](#SectionIII) |  | Expanded statistical analysis | 31 |

***Section I*: Expanded results**

**Table S1.** Iron homeostasis gene, data extraction, and extreme outlier summary

| Gene Symbol | Gene Name | Extraction window^a^ | Number of CpG sites | Data status^b^ | Extreme Outliers^c^ |
| --- | --- | --- | --- | --- | --- |
| *ACO1* | Iron regulatory protein 1 | chr9:32382601-32452832 | 11 | Available | 31 |
| *ACO2* | Iron regulatory protein 2 | chr22:41863129-41926993 | 12 | Available | 60 |
| *APP^d^* | Amyloid precursor protein | chr21:27250861-27545138 | 21 | Available | 62 |
| *CALR* | Calreticulin | chr19:13047414-13057304 | 30 | Available | 39 |
| *CD163* | Hemoglobin scavenger receptor | chr12:7621412-7658414 | 3 | Available | 38 |
| *CP* | Ceruloplasmin | chr3:148888290-148941832 | 6 | Available | 20 |
| *CUBN* | Cubilin | chr10:16863965-17173816 | 15 | Available | 50 |
| *CYBRD1* | Duodenal cytochrome b | chr2:172376866-172416643 | 13 | Available | 31 |
| *FECH* | Ferrochelatase | chr18:55210073-55255969 | 13 | Available | 30 |
| *FLVCR1* | Feline leukemia virus subgroup C receptor | chr1:213029597-213074705 | 18 | Available | 28 |
| *FTH1* | Ferritin heavy | chr11:61729757-61737132 | 20 | Available | 35 |
| *FTL* | Ferritin light | chr19:49466566-49472136 | 11 | Available | 25 |
| *FTMT* | Mitochondrial ferritin | chr5:121185650-121190523 | 8 | Available | 28 |
| *FXN* | Frataxin | chr9:71648479-71695993 | 13 | Available | 31 |
| *GSTP1* | Glutathione S Transferase | chr11:67349066-67356124 | 12 | Available | 28 |
| *GLRX5* | Glutaredoxin 5 |  | 16 | Available | 52 |
| *HAMP* | Hepcidin | chr19:35771410-35778045 | NA | Analyzed in pilot work^1^ | NA |
| *HEPH* | Hephaestin | chrX:65380433-65489230 | NA | Data not available | NA |
| *HFE* | Human hemochromatosis protein | chr6:26094615-26099056 | 1 | Available | 0 |
| *HJV (HFE2)* | Hemojuvelin BMP co-Receptor | chr1:145411191-145419545 | 13 | Available | 28 |
| HMOX1 | Heme-oxygenase 1 | chr22:35775060-35792207 | 8 | Available | 29 |
| HMOX2 | Heme-oxygenase 2 | chr16:4524341-4562348 | 22 | Available | 61 |
| *HP* | Haptoglobin | chr16:72086508-72096955 | 5 | Available | 15 |
| *HPX* | Hemopexin | chr11:6450268-6464254 | 7 | Available | 23 |
| *IREB2* | Iron responsive element binding | chr15:78728518-78795798 | 15 | Available | 36 |
| *LRP1* | LDL receptor related protein | chr12:57520282-57609125 | 91 | Available | 189 |
| *PCBP1* | poly(RC) Binding Protein 1 | chr2:70312585-70318334 | 17 | Available | 52 |
| *PGRMC1* | Progesterone receptor membrane | chrX:118368211-118380429 | NA | Data not available | NA |
| *SLC46A1* | Heme carrying protein 1 | chr17:26719661-26735230 | 15 | Available | 39 |
| *SLC11A1* | Solute Carrier Family 11 Member 1 | chr2:219244752-219263617 | 26 | Available | 45 |
| *SLC11A2* | Divalent metal transporter 1 | chr12:51377775-51424058 | 15 | Available | 29 |
| *SLC25A37* | Solute Carrier Family 25 Member 37 (Mitoferrin 1) | chr8:23384363-23432063 | 28 | Available | 61 |
| *SLC40A1 (FP)* | Solute Carrier Family 40 Member 1 (Ferroportin) | chr2:190423316-190447537 | 14 | Available | 43 |
| *SLC48A1* | Solute carrier family 48 member 1 | chr12:48164967-48178536 | 14 | Available | 43 |
| *STEAP3^d^* | STEAP3 Metalloreductase | chr2:119979384-120025227 | 29 | Available | 51 |
| *TF* | Transferrin | chr3:133462800-133499850 | 15 | Available | 57 |
| *TFRC* | Transferrin receptor 1 | chr3:195774155-195811032 | 16 | Available | 43 |
| *TFR2* | Transferrin receptor 2 | chr7:100216039-100241201 | 29 | Available | 60 |
| *TNF^d^* | Tumor necrosis factor | chr6:31542292-31548112 | 35 | Available | 62 |

^a^Window for discovery phase data extraction from the genome-wide DNA methylation data, positions based on UCSC Genome Browser Build GRCh37/hg19, transcription start/stop location; ^b^Data status column depicts if discovery phase DNA methylation data were available for a gene of interest (Available, analyzed as part of currently study; Data not available, data removed as part of QC pipeline due to sex chromosome location); ^c^Number of extreme DNA methylation outliers (three times the interquartile range) score adjusted and pulled in for the discovery phase data; ^d^CpG sites within these genes were ultimately examined during the replication phase

**Table S2.** Number of participants with DNA methylation data by day in discovery and replication samples

| Day | Discovery (n=260) | Replication (n=100) |
| --- | --- | --- |
| 1 | 62 | 25 |
| 2 | 89 | 26 |
| 3 | 52 | 22 |
| 4 | 86 | 24 |
| 5 | 103 | 34 |
| 6 | 52 | 29 |
| 7 | 80 | 24 |
| 8 | 84 | 24 |
| 9 | 33 | 21 |
| 10 | 69 | 27 |
| 11 | 51 | 18 |
| 12 | 32 | 8 |
| 13 | 46 | 15 |
| 14 | 0 | 6 |

DNA methylation trajectories were inferred for 260 and 100 participants in the discovery and replication analyses, respectively; longitudinal CSF DNAm data were available for participants over 13 and 14 days post-injury in the discovery and replication analyses, respectively; participants had between 2 and 5 DNAm observations with an average of 3.2 observations in our discovery sample and between 2 and 4 DNAm observations with an average of 3.0 observations in our replication sample

**Table S3.** Summary of significant and suggestive associations for base models (no adjustment for CTH)

| Outcome | Gene | CpG | Position^a^ | UCSC Region^b^ | Relationship to Island^c^ | *p* |
| --- | --- | --- | --- | --- | --- | --- |
| GOS-12 | *APP* | cg08866780 | 27543523 | TSS1500 | Island | 0.00013 |
| GOS-3 | *TNF* | cg08553327 | 31543647 | 1stExon | OpenSea | 0.00027 |
| GOS-3 | *TNF* | cg21222743 | 31543545 | 1stExon | OpenSea | 0.00032 |
| GOS-3 | *TNF* | cg10717214 | 31543557 | 1stExon | OpenSea | 0.00039 |
| GOS-3 | *TNF* | cg08553327 | 31543647 | 1stExon | OpenSea | 0.00055 |
| Death-12 | *APP* | cg08866780 | 27543523 | TSS1500 | Island | 0.00058 |
| GOS-3 | *STEAP3* | cg25713625 | 120022835 | 3'UTR | S_Shore | 0.00061 |
| GOS-3 | *SLC25A37* | cg18186478 | 23423960 | Body | OpenSea | 0.00061 |
| Death-12 | *ACO1* | cg13977526 | 32384244 | TSS1500 | N_Shore | 0.00062 |
| GOS-3 | *TNF* | cg10650821 | 31543686 | 1stExon | OpenSea | 0.00083 |
| GOS-3 | *TNF* | cg12681001 | 31543540 | 1stExon | OpenSea | 0.00089 |
| Death-3 | *APP* | cg08866780 | 27543523 | TSS1500 | Island | 0.00099 |
| GO3 | *LRP1* | cg09150519 | 57566995 | Body | N_Shelf | 0.00116 |
| GOS-3 | *FECH* | cg15864104 | 55254508 | TSS1500 | S_Shore | 0.00118 |
| GOS-3 | *TNF* | cg21222743 | 31543545 | 1stExon | OpenSea | 0.00121 |
| GOS-3 | *FTH1* | cg24803614 | 61733475 | Body | N_Shore | 0.00125 |
| GOS-3 | *FXN* | cg02268013 | 71650331 | TSS200 | N_Shore | 0.00133 |
| GOS-3 | *FXN* | cg21229044 | 71669714 | Body | OpenSea | 0.00151 |
| GOS-12 | *LRP1* | cg12069468 | 57578649 | Body | N_Shore | 0.00155 |
| GOS-12 | *STEAP3* | cg25713625 | 120022835 | 3'UTR | S_Shore | 0.00182 |
| GOS-3 | *FLVCR1* | cg23082281 | 213032677 | TSS1500;Body | S_Shore | 0.00184 |
| GOS-3 | *SLC11A2* | cg22826226 | 51421367 | TSS1500 | S_Shore | 0.00192 |
| GOS-3 | *LRP1* | cg12069468 | 57578649 | Body | N_Shore | 0.00223 |
| Death-12 | *TNF* | cg10717214 | 31543557 | 1stExon | OpenSea | 0.00251 |
| Death-12 | *LRP1* | cg12069468 | 57578649 | Body | N_Shore | 0.00273 |
| GOS-3 | *HMOX2* | cg14951292 | 4525986 | TSS1500;5'UTR | N_Shore | 0.00289 |
| Death-3 | *ACO2* | cg25636833 | 41864805 | TSS200;TSS1500 | Island | 0.00311 |
| GOS-3 | *TNF* | cg21467614 | 31543638 | 1stExon | OpenSea | 0.00316 |
| Death-12 | *TNF* | cg21222743 | 31543545 | 1stExon | OpenSea | 0.00368 |
| Death-12 | *TNF* | cg08553327 | 31543647 | 1stExon | OpenSea | 0.00370 |
| GOS-3 | *HFE2* | cg26036288 | 145411967 | TSS1500 | N_Shelf | 0.00388 |
| GOS-3 | *CYBRD1* | cg15691325 | 172387940 | Body | OpenSea | 0.00399 |
| Death-12 | *STEAP3* | cg25713625 | 120022835 | 3'UTR | S_Shore | 0.00410 |
| GOS-12 | *APP* | cg03779431 | 27543124 | 1stExon;5'UTR | Island | 0.00411 |
| Death-3 | *LRP1* | cg09150519 | 57566995 | Body | N_Shelf | 0.00461 |
| GOS-12 | *ACO1* | cg13977526 | 32384244 | TSS1500 | N_Shore | 0.00502 |
| GOS-3 | *LRP1* | cg22339313 | 57578642 | Body | N_Shore | 0.00512 |
| GOS-12 | *HMOX2* | cg14951292 | 4525986 | TSS1500 | N_Shore | 0.00520 |
| GOS-3 | *APP* | cg08866780 | 27543523 | TSS1500 | Island | 0.00596 |
| Death-12 | *IREB2* | cg08092050 | 78734858 | Body | S_Shelf | 0.00621 |
| GOS-3 | *IREB2* | cg03700171 | 78748272 | Body | OpenSea | 0.00628 |
| GOS-12 | *TFRC* | cg09470983 | 195805948 | 5'UTR | N_Shelf | 0.00639 |
| GOS-3 | *TF* | cg20123637 | 133467435 | Body | S_Shelf | 0.00672 |
| GOS-3 | *IREB2* | cg08092050 | 78734858 | Body | S_Shelf | 0.00714 |
| Death-12 | *LRP1* | cg06531661 | 57522120 | TSS200 | Island | 0.00722 |
| Death-3 | *FLVCR1* | cg23082281 | 213032677 | TSS1500;Body | S_Shore | 0.00729 |
| Death-12 | *LRP1* | cg16612926 | 57601671 | Body | OpenSea | 0.00779 |
| GOS-12 | *FXN* | cg21229044 | 71669714 | Body | OpenSea | 0.00791 |
| Death-12 | *FXN* | cg21229044 | 71669714 | Body | OpenSea | 0.00800 |
| GOS-3 | *GSTP1* | cg06928838 | 67351490 | Body | Island | 0.00826 |
| Death-3 | *TNF* | cg10717214 | 31543557 | 1stExon | OpenSea | 0.00828 |
| Death-12 | *CALR* | cg27180563 | 13056485 | TSS200 | Island | 0.00835 |
| DCI | *TF* | cg16262614 | 133464971 | TSS200 | Island | 0.00840 |
| Death-12 | *TNF* | cg12681001 | 31543540 | 1stExon | OpenSea | 0.00851 |
| GOS-3 | *TFRC* | cg23502966 | 195789749 | Body | OpenSea | 0.00875 |
| Death-12 | *SLC25A37* | cg00892891 | 23423734 | Body | OpenSea | 0.00877 |
| CV | *FXN* | cg07158339 | 71650237 | TSS1500 | N_Shore | 0.00880 |
| Death-3 | *GLRX5* | cg01304278 | 96001998 | Body;TSS1500 | S_Shore | 0.00886 |
| Death-3 | *TNF* | cg08553327 | 31543647 | 1stExon | OpenSea | 0.00892 |
| Death-12 | *LRP1* | cg09150519 | 57566995 | Body | N_Shelf | 0.00931 |
| Death-3 | *STEAP3* | cg25713625 | 120022835 | 3'UTR | S_Shore | 0.00933 |
| Death-3 | *TNF* | cg21222743 | 31543545 | 1stExon | OpenSea | 0.00948 |
| Death-3 | *FXN* | cg21229044 | 71669714 | Body | OpenSea | 0.00959 |
| Death-3 | *FXN* | cg02268013 | 71650331 | TSS200 | N_Shore | 0.00961 |
| GOS-3 | *CP* | cg05776336 | 148939487 | 1stExon | OpenSea | 0.00983 |
| GOS-3 | *CP* | cg24876069 | 148916713 | Body | OpenSea | 0.00992 |
| GOS-3 | *LRP1* | cg11813198 | 57572413 | Body | S_Shelf | 0.01039 |
| GOS-3 | *TFRC* | cg09470983 | 195805948 | 5'UTR | N_Shelf | 0.01040 |
| GOS-12 | *GLRX5* | cg01304278 | 96001998 | Body;TSS1500 | S_Shore | 0.01049 |
| Death-12 | *SLC11A2* | cg22826226 | 51421367 | TSS1500 | S_Shore | 0.01065 |
| GOS-12 | *SLC25A37* | cg18186478 | 23423960 | Body | OpenSea | 0.01073 |
| GOS-3 | *CP* | cg19278448 | 148905466 | Body | OpenSea | 0.01078 |
| GOS-3 | *LRP1* | cg21702971 | 57588243 | Body;TSS200 | OpenSea | 0.01107 |
| GOS-12 | *GLRX5* | cg02940042 | 96000874 | TSS1500;Body | Island | 0.01144 |
| Death-3 | *GSTP1* | cg06928838 | 67351490 | Body | Island | 0.01164 |
| Death-3 | *TF* | cg20123637 | 133467435 | Body | S_Shelf | 0.01207 |
| Death-12 | *GLRX5* | cg02940042 | 96000874 | TSS1500;Body | Island | 0.01252 |
| Death-3 | *ACO1* | cg13977526 | 32384244 | TSS1500 | N_Shore | 0.01293 |
| GOS-3 | *LRP1* | cg16612926 | 57601671 | Body | OpenSea | 0.01344 |
| GOS-3 | *GLRX5* | cg02940042 | 96000874 | TSS1500;Body | Island | 0.01356 |
| Death-3 | *IREB2* | cg08092050 | 78734858 | Body | S_Shelf | 0.01381 |
| GOS-12 | *LRP1* | cg22339313 | 57578642 | Body | N_Shore | 0.01388 |
| GOS-12 | *TNF* | cg10717214 | 31543557 | 1stExon | OpenSea | 0.01388 |
| GOS-3 | *CYBRD1* | cg15691325 | 172387940 | Body;Body | OpenSea | 0.01395 |
| CV | *SLC25A37* | cg24126361 | 23398346 | Body | OpenSea | 0.01451 |
| Death-12 | *SLC25A37* | cg18186478 | 23423960 | Body | OpenSea | 0.01458 |
| Death-3 | *FECH* | cg17299799 | 55214063 | 3'UTR | OpenSea | 0.01491 |
| Death-12 | *FXN* | cg02268013 | 71650331 | TSS200 | N_Shore | 0.01493 |
| GOS-12 | *HPX* | cg15741583 | 6461908 | Body | OpenSea | 0.01513 |
| Death-3 | *GLRX5* | cg02940042 | 96000874 | TSS1500;Body | Island | 0.01514 |
| GOS-12 | *LRP1* | cg09150519 | 57566995 | Body | N_Shelf | 0.01551 |
| GOS-3 | *IREB2* | cg13714459 | 78730363 | TSS200 | Island | 0.01571 |
| GOS-3 | *CALR* | cg27180563 | 13056485 | TSS200 | Island | 0.01575 |
| DCI | *CYBRD1* | cg15691325 | 172387940 | Body | OpenSea | 0.01581 |
| GOS-3 | *LRP1* | cg06531661 | 57522120 | TSS200 | Island | 0.01586 |
| CV | *ACO2* | cg25636833 | 41864805 | TSS200;TSS1500 | Island | 0.01604 |
| DCI | *FLVCR1* | cg18789758 | 213030449 | Body;TSS1500 | N_Shore | 0.01622 |
| Death-3 | *FP* | cg10752008 | 190445175 | 1stExon | N_Shore | 0.01625 |
| GOS-3 | *SLC48A1* | cg01154392 | 48167362 | Body | Island | 0.01656 |
| Death-3 | *FECH* | cg26043149 | 55253948 | 5'UTR;1stExon | Island | 0.01669 |
| GOS-3 | *TNF* | cg12681001 | 31543540 | 1stExon | OpenSea | 0.01692 |
| Death-3 | *SLC11A2* | cg22826226 | 51421367 | TSS1500 | S_Shore | 0.01696 |
| GOS-3 | *FLVCR1* | cg18789758 | 213030449 | Body;TSS1500 | N_Shore | 0.01718 |
| Death-12 | *GLRX5* | cg01304278 | 96001998 | Body;TSS1500 | S_Shore | 0.01737 |
| GOS-12 | *LRP1* | cg06531661 | 57522120 | TSS200 | Island | 0.01744 |
| Death-12 | *ACO2* | cg25636833 | 41864805 | TSS200;TSS1500 | Island | 0.01783 |
| Death-3 | *PCBP1* | cg26487157 | 70313295 | TSS1500 | Island | 0.01808 |
| Death-3 | *TNF* | cg12681001 | 31543540 | 1stExon | OpenSea | 0.01827 |
| Death-3 | *LRP1* | cg16612926 | 57601671 | Body | OpenSea | 0.01835 |
| GOS-3 | *APP* | cg15407086 | 27543045 | 1stExon;5'UTR | Island | 0.01839 |
| CV | *CALR* | cg27180563 | 13056485 | TSS200 | Island | 0.01842 |
| Death-12 | *FXN* | cg02268013 | 71650331 | TSS200 | N_Shore | 0.01876 |
| DCI | *SLC25A37* | cg24917065 | 23418389 | Body | OpenSea | 0.01880 |
| GOS-3 | *FXN* | cg14410042 | 71654823 | Body | S_Shelf | 0.01905 |
| GOS-12 | *TFRC* | cg21494636 | 195808986 | 1stExon;5'UTR;TSS200 | Island | 0.01955 |
| GOS-3 | *HFE2* | cg00987513 | 145413337 | 1stExon;5'UTR | N_Shelf | 0.01966 |
| GOS-12 | *TNF* | cg21222743 | 31543545 | 1stExon | OpenSea | 0.01986 |
| Death-12 | *TNF* | cg21467614 | 31543638 | 1stExon | OpenSea | 0.02027 |
| GOS-3 | *FLVCR1* | cg14912380 | 213068483 | Body | OpenSea | 0.02041 |
| Death-12 | *TNF* | cg10650821 | 31543686 | 1stExon | OpenSea | 0.02068 |
| GOS-12 | *TNF* | cg08553327 | 31543647 | 1stExon | OpenSea | 0.02086 |
| Death-12 | *FLVCR1* | cg18789758 | 213030449 | Body;TSS1500 | N_Shore | 0.02094 |
| Death-3 | *TF* | cg20123637 | 133467435 | Body | S_Shelf | 0.02094 |
| Death-12 | *LRP1* | cg22339313 | 57578642 | Body | N_Shore | 0.02131 |
| Death-3 | *LRP1* | cg12069468 | 57578649 | Body | N_Shore | 0.02135 |
| GOS-12 | *ACO2* | cg25636833 | 41864805 | TSS200;TSS1500 | Island | 0.02164 |
| Death-12 | *TFR2* | cg19767562 | 100224437 | Body | Island | 0.02181 |
| CV | *ACO2* | cg26423539 | 41864790 | TSS200;TSS1500 | Island | 0.02209 |
| Death-12 | *TNF* | cg21222743 | 31543545 | 1stExon | OpenSea | 0.02218 |
| GOS-3 | *PCBP1* | cg00396520 | 70314546 | TSS200 | Island | 0.02226 |
| GOS-3 | *HPX* | cg15741583 | 6461908 | Body | OpenSea | 0.02284 |
| DCI | *HMOX2* | cg06605704 | 4551147 | 5'UTR | OpenSea | 0.02351 |
| Death-12 | *TNF* | cg08553327 | 31543647 | 1stExon | OpenSea | 0.02433 |
| CV | *CYBRD1* | cg15691325 | 172387940 | Body | OpenSea | 0.02464 |
| Death-12 | *GSTP1* | cg06928838 | 67351490 | Body | Island | 0.02538 |
| Death-12 | *CP* | cg24876069 | 148916713 | Body | OpenSea | 0.02546 |
| Death-3 | *FLVCR1* | cg18789758 | 213030449 | Body;TSS1500 | N_Shore | 0.02563 |
| Death-3 | *TNF* | cg08553327 | 31543647 | 1stExon | OpenSea | 0.02573 |
| Death-12 | *FTL* | cg05617973 | 49468917 | Body | Island | 0.02588 |
| Death-12 | *FTH1* | cg04230589 | 61735144 | TSS200 | Island | 0.02601 |
| GOS-12 | *CP* | cg19278448 | 148905466 | Body | OpenSea | 0.02602 |
| DCI | *FTH1* | cg22892043 | 61731930 | 3'UTR | N_Shelf | 0.02668 |
| Death-3 | *IREB2* | cg13714459 | 78730363 | TSS200 | Island | 0.02694 |
| GOS-3 | *TNF* | cg02581828 | 31546769 |  | N_Shore | 0.02704 |
| GOS-12 | *SLC11A2* | cg22826226 | 51421367 | TSS1500 | S_Shore | 0.02744 |
| Death-12 | *FP* | cg10752008 | 190445175 | 1stExon | N_Shore | 0.02745 |
| GOS-12 | *LRP1* | cg05943813 | 57573255 | Body | S_Shelf | 0.02790 |
| GOS-12 | *LRP1* | cg21702971 | 57588243 | Body;TSS200 | OpenSea | 0.02828 |
| CV | *FTH1* | cg04230589 | 61735144 | TSS200 | Island | 0.02848 |
| GOS-3 | *LRP1* | cg14621254 | 57569787 | Body | Island | 0.02880 |
| GOS-12 | *TFR2* | cg19767562 | 100224437 | Body | Island | 0.02889 |
| DCI | *TNF* | cg12681001 | 31543540 | 1stExon | OpenSea | 0.02892 |
| GOS-3 | *HFE2* | cg14036143 | 145415441 | 5'UTR;Body | Island | 0.02895 |
| GOS-12 | *LRP1* | cg06531661 | 57522120 | TSS200 | Island | 0.02900 |
| GOS-12 | *STEAP3* | cg25713625 | 120022835 | 3'UTR | S_Shore | 0.02915 |
| Death-12 | *TF* | cg17775713 | 133465469 | Body | S_Shore | 0.02943 |
| GOS-12 | *FTL* | cg26532042 | 49468557 | TSS200 | Island | 0.02987 |
| GOS-3 | *FTH1* | cg02315732 | 61732658 | Body | N_Shelf | 0.02988 |
| GOS-12 | *SLC48A1* | cg01154392 | 48167362 | Body | Island | 0.03037 |
| GOS-12 | *TFR2* | cg19767562 | 100224437 | Body | Island | 0.03048 |
| GOS-3 | *SLC11A2* | cg25493658 | 51421225 | TSS1500 | S_Shore | 0.03077 |
| Death-3 | *TNF* | cg21222743 | 31543545 | 1stExon | OpenSea | 0.03087 |
| GOS-12 | *LRP1* | cg10904028 | 57590920 | Body | OpenSea | 0.03137 |
| Death-12 | *TF* | cg24512138 | 133465360 | Body | Island | 0.03140 |
| Death-12 | *STEAP3* | cg25508118 | 119981769 | 5'UTR | Island | 0.03148 |
| GOS-3 | *ACO2* | cg25636833 | 41864805 | TSS200;TSS1500 | Island | 0.03202 |
| GOS-12 | *FTH1* | cg04230589 | 61735144 | TSS200 | Island | 0.03212 |
| CV | *CUBN* | cg17244673 | 17166078 | Body | OpenSea | 0.03218 |
| GOS-3 | *FLVCR1* | cg23082281 | 213032677 | TSS1500;Body | S_Shore | 0.03254 |
| GOS-3 | *CYBRD1* | cg24200501 | 172378036 | TSS1500 | N_Shore | 0.03284 |
| CV | *CP* | cg17439694 | 148939523 | 1stExon | OpenSea | 0.03322 |
| Death-12 | *TF* | cg20123637 | 133467435 | Body | S_Shelf | 0.03331 |
| Death-12 | *APP* | cg23269692 | 27372446 | Body | OpenSea | 0.03349 |
| GOS-3 | *LRP1* | cg22339313 | 57578642 | Body | N_Shore | 0.03372 |
| GOS-3 | *TNF* | cg10717214 | 31543557 | 1stExon | OpenSea | 0.03491 |
| Death-12 | *TF* | cg24512138 | 133465360 | Body | Island | 0.03494 |
| GOS-12 | *TNF* | cg23204396 | 31548107 |  | N_Shore | 0.03500 |
| GOS-3 | *FTMT* | cg09441819 | 121186479 | TSS1500 | N_Shore | 0.03557 |
| Death-3 | *CP* | cg19278448 | 148905466 | Body | OpenSea | 0.03566 |
| GOS-3 | *APP* | cg15407086 | 27543045 | 1stExon;5'UTR | Island | 0.03570 |
| Death-12 | *LRP1* | cg01276169 | 57569898 | Body | Island | 0.03572 |
| GOS-3 | *FXN* | cg02268013 | 71650331 | TSS200 | N_Shore | 0.03594 |
| Death-3 | *LRP1* | cg01276169 | 57569898 | Body | Island | 0.03616 |
| GOS-3 | *TF* | cg18286850 | 133464713 | TSS1500 | N_Shore | 0.03625 |
| DCI | *TNF* | cg21467614 | 31543638 | 1stExon | OpenSea | 0.03650 |
| GOS-3 | *TNF* | cg26729380 | 31543655 | 1stExon | OpenSea | 0.03664 |
| Death-12 | *FECH* | cg17299799 | 55214063 | 3'UTR | OpenSea | 0.03709 |
| CV | *GSTP1* | cg14921275 | 67350511 | TSS1500 | N_Shore | 0.03710 |
| Death-3 | *TNF* | cg10650821 | 31543686 | 1stExon | OpenSea | 0.03717 |
| GOS-12 | *IREB2* | cg08092050 | 78734858 | Body | S_Shelf | 0.03767 |
| CV | *SLC25A37* | cg24126361 | 23398346 | Body | OpenSea | 0.03841 |
| DCI | *CUBN* | cg17436460 | 17028480 | Body | OpenSea | 0.03879 |
| DCI | *TNF* | cg09637172 | 31545252 | Body | N_Shelf | 0.03914 |
| DCI | *TNF* | cg10717214 | 31543557 | 1stExon | OpenSea | 0.03964 |
| GOS-12 | *STEAP3* | cg25508118 | 119981769 | 5'UTR | Island | 0.03970 |
| GOS-3 | *SLC25A37* | cg20845639 | 23430886 |  | S_Shore | 0.04010 |
| GOS-12 | *CALR* | cg27180563 | 13056485 | TSS200 | Island | 0.04023 |
| CV | *CP* | cg05776336 | 148939487 | 1stExon | OpenSea | 0.04023 |
| CV | *FTH1* | cg04230589 | 61735144 | TSS200 | Island | 0.04027 |
| Death-3 | *FXN* | cg02268013 | 71650331 | TSS200 | N_Shore | 0.04030 |
| Death-12 | *HMOX2* | cg14951292 | 4525986 | TSS1500 | N_Shore | 0.04036 |
| GOS-3 | *SLC46A1* | cg00497630 | 26733052 | 1stExon | Island | 0.04041 |
| GOS-12 | *SLC25A37* | cg00892891 | 23423734 | Body | OpenSea | 0.04052 |
| Death-12 | *TFR2* | cg19767562 | 100224437 | Body | Island | 0.04113 |
| Death-12 | *TNF* | cg26729380 | 31543655 | 1stExon | OpenSea | 0.04124 |
| DCI | *TF* | cg08234618 | 133464842 | TSS200 | N_Shore | 0.04133 |
| Death-12 | *FECH* | cg26043149 | 55253948 | 5'UTR;1stExon | Island | 0.04148 |
| Death-12 | *LRP1* | cg12146864 | 57569768 | Body | Island | 0.04198 |
| CV | *PCBP1* | cg09175843 | 70313417 | TSS1500 | Island | 0.04228 |
| Death-3 | *TNF* | cg21467614 | 31543638 | 1stExon | OpenSea | 0.04280 |
| GOS-12 | *HFE2* | cg00987513 | 145413337 | 5'UTR;1stExon | N_Shelf | 0.04306 |
| Death-12 | *TF* | cg20123637 | 133467435 | Body | S_Shelf | 0.04324 |
| GOS-12 | *TNF* | cg21222743 | 31543545 | 1stExon | OpenSea | 0.04361 |
| DCI | *GLRX5* | cg12096759 | 96002581 | Body;TSS1500 | S_Shore | 0.04390 |
| GOS-3 | *FLVCR1* | cg00156403 | 213035301 | Body | S_Shelf | 0.04418 |
| GOS-12 | *IREB2* | cg13714459 | 78730363 | TSS200 | Island | 0.04541 |
| Death-12 | *FTH1* | cg19407570 | 61735476 | TSS1500 | Island | 0.04581 |
| GOS-12 | *LRP1* | cg16612926 | 57601671 | Body | OpenSea | 0.04611 |
| DCI | *TF* | cg16262614 | 133464971 | TSS200 | Island | 0.04632 |
| Death-12 | *FLVCR1* | cg14912380 | 213068483 | Body | OpenSea | 0.04633 |
| GOS-12 | *FTL* | cg26532042 | 49468557 | TSS200 | Island | 0.04639 |
| GOS-3 | *CP* | cg17439694 | 148939523 | 1stExon | OpenSea | 0.04651 |
| Death-3 | *SLC25A37* | cg18186478 | 23423960 | Body | OpenSea | 0.04657 |
| Death-3 | *LRP1* | cg06531661 | 57522120 | TSS200 | Island | 0.04681 |
| Death-3 | *CP* | cg19278448 | 148905466 | Body | OpenSea | 0.04689 |
| GOS-12 | *TNF* | cg12681001 | 31543540 | 1stExon | OpenSea | 0.04730 |
| GOS-3 | *HFE2* | cg14036143 | 145415441 | 5'UTR;Body | Island | 0.04739 |
| Death-3 | *TNF* | cg26729380 | 31543655 | 1stExon | OpenSea | 0.04789 |
| GOS-12 | *TF* | cg20123637 | 133467435 | Body | S_Shelf | 0.04794 |
| GOS-12 | *CALR* | cg27180563 | 13056485 | TSS200 | Island | 0.04813 |
| GOS-3 | *TF* | cg16267236 | 133464463 | TSS1500 | N_Shore | 0.04846 |
| Death-12 | *LRP1* | cg10904028 | 57590920 | Body | OpenSea | 0.04856 |
| Death-3 | *FTH1* | cg19407570 | 61735476 | TSS1500 | Island | 0.04877 |
| CV | *CP* | cg09457255 | 148933841 | Body | OpenSea | 0.04934 |
| Death-12 | *CP* | cg19278448 | 148905466 | Body | OpenSea | 0.04954 |

Binary logistic regression associations of base model (i.e., no adjustment for CTH) trajectory group with patient outcomes while controlling for age, sex, self-identified race, and Fisher grade; associations with p-values <0.05 presented here; table sorted by p-value; CV, Cerebral vasospasm; DCI, Delayed cerebral ischemia; GOS-3, Glasgow Outcome Scale at 3 months; GOS-12, Glasgow Outcome Scale at 12 months; Death-3, death at 3 months; Death-12, death at 12 months; ^a^UCSC Genome Browser Build GRCh37/hg19; ^b^Trimmed to unique listings, multiple listings indicate information for multiple transcripts; TSS, transcription start site; TSS200 = 0-200 bases upstream of the TSS; TSS1500 = 200-1500 bases upstream of the TSS; 5'UTR = 5' untranslated region, between the TSS and the ATG start site; Body = Between the ATG and stop codon; irrespective of the presence of introns, exons, TSS, or promoters; 3'UTR = between the stop codon and poly A tail; ^c^Shore, 0-2 kb from island; Shelf, 2-4 kb from island; N, upstream (5') of CpG island; S, downstream (3') of CpG island

**Table S4.** Summary of significant and suggestive associations for CTH-adjusted models

| Outcome | Gene | CpG | Position^a^ | UCSC Region^b^ | Relationship to Island^c^ | *p* |
| --- | --- | --- | --- | --- | --- | --- |
| GOS-3 | *SLC46A1* | cg00497630 | 26733052 | 1stExon | Island | 0.00066 |
| Death-3 | *ACO2* | cg25636833 | 41864805 | TSS200;TSS1500 | Island | 0.00088 |
| Death-12 | *APP* | cg23269692 | 27372446 | Body | OpenSea | 0.00337 |
| CV | *SLC40A1* | cg19075346 | 190445196 | 5'UTR;1stExon | N_Shore | 0.00368 |
| Death-3 | *FLVCR1* | cg23082281 | 213032677 | TSS1500;Body | S_Shore | 0.00385 |
| GOS-3 | *LRP1* | cg21702971 | 57588243 | Body;TSS200 | OpenSea | 0.00392 |
| GOS-3 | *APP* | cg08866780 | 27543523 | TSS1500 | Island | 0.00399 |
| DCI | *PCBP1* | cg19638749 | 70312615 |  | N_Shore | 0.00401 |
| DCI | *TFR2* | cg12792367 | 100238750 | Body | OpenSea | 0.00404 |
| Death-3 | *SLC11A2* | cg01043320 | 51419821 | 5'UTR | Island | 0.00433 |
| Death-12 | *ACO2* | cg25636833 | 41864805 | TSS200;TSS1500 | Island | 0.00453 |
| Death-12 | *FTH1* | cg19407570 | 61735476 | TSS1500 | Island | 0.00469 |
| GOS-3 | *SLC11A2* | cg01043320 | 51419821 | 5'UTR | Island | 0.00473 |
| GOS-12 | *ACO2* | cg25636833 | 41864805 | TSS200;TSS1500 | Island | 0.00493 |
| DCI | *SLC40A1* | cg15599483 | 190442823 | Body | N_Shelf | 0.00495 |
| GOS-12 | *STEAP3* | cg12535642 | 120020901 | Body | Island | 0.00515 |
| GOS-3 | *TNF* | cg21467614 | 31543638 | 1stExon | OpenSea | 0.00578 |
| GOS-3 | *TNF* | cg24540603 | 31547917 |  | N_Shore | 0.00739 |
| GOS-12 | *APP* | cg08866780 | 27543523 | TSS1500 | Island | 0.00968 |
| Death-3 | *FTH1* | cg19407570 | 61735476 | TSS1500 | Island | 0.00971 |
| GOS-12 | *STEAP3* | cg06872331 | 119981960 | 5'UTR | S_Shore | 0.01014 |
| CV | *SLC25A37* | cg24126361 | 23398346 | Body | OpenSea | 0.01040 |
| GOS-3 | *LRP1* | cg10233454 | 57529389 | Body | OpenSea | 0.01052 |
| CV | *CYBRD1* | cg24200501 | 172378036 | TSS1500 | N_Shore | 0.01057 |
| Death-12 | *IREB2* | cg07989511 | 78730357 | TSS200 | Island | 0.01170 |
| GOS-3 | *FTH1* | cg24803614 | 61733475 | Body | N_Shore | 0.01268 |
| GOS-3 | *TNF* | cg12681001 | 31543540 | 1stExon | OpenSea | 0.01274 |
| Death-12 | *APP* | cg08866780 | 27543523 | TSS1500 | Island | 0.01284 |
| GOS-3 | *TNF* | cg15989608 | 31545321 | 3'UTR | N_Shelf | 0.01347 |
| GOS-3 | *LRP1* | cg22339313 | 57578642 | Body | N_Shore | 0.01387 |
| Death-12 | *SLC11A2* | cg01043320 | 51419821 | 5'UTR | Island | 0.01453 |
| GOS-3 | *FTL* | cg12026095 | 49468461 | TSS200 | Island | 0.01543 |
| Death-3 | *APP* | cg23269692 | 27372446 | Body | OpenSea | 0.01563 |
| CV | *TNF* | cg14306709 | 31547704 |  | N_Shore | 0.01595 |
| GOS-3 | *ACO2* | cg25636833 | 41864805 | TSS200;TSS1500 | Island | 0.01621 |
| Death-12 | *FTL* | cg05617973 | 49468917 | Body | Island | 0.01761 |
| GOS-12 | *STEAP3* | cg25713625 | 120022835 | 3'UTR | S_Shore | 0.01817 |
| Death-12 | *STEAP3* | cg06872331 | 119981960 | 5'UTR | S_Shore | 0.01974 |
| CV | *TNF* | cg15989608 | 31545321 | 3'UTR | N_Shelf | 0.02050 |
| GOS-3 | *TNF* | cg21370522 | 31543219 | TSS200 | OpenSea | 0.02071 |
| Death-12 | *SLC11A1* | cg07719512 | 219246576 | TSS200 | OpenSea | 0.02242 |
| GOS-3 | *GSTP1* | cg11566244 | 67351786 | Body | Island | 0.02335 |
| GOS-3 | *FLVCR1* | cg23082281 | 213032677 | TSS1500;Body | S_Shore | 0.02364 |
| Death-3 | *APP* | cg08866780 | 27543523 | TSS1500 | Island | 0.02366 |
| Death-12 | *STEAP3* | cg25508118 | 119981769 | 5'UTR | Island | 0.02481 |
| CV | *CYBRD1* | cg15691325 | 172387940 | Body | OpenSea | 0.02535 |
| DCI | *TF* | cg08234618 | 133464842 | TSS200 | N_Shore | 0.02601 |
| GOS-12 | *SLC46A1* | cg00497630 | 26733052 | 1stExon | Island | 0.02626 |
| Death-12 | *FLVCR1* | cg23082281 | 213032677 | TSS1500;Body | S_Shore | 0.02628 |
| CV | *HFE2* | cg14036143 | 145415441 | 5'UTR;Body | Island | 0.02667 |
| DCI | *HFE2* | cg14036143 | 145415441 | 5'UTR;Body | Island | 0.02681 |
| GOS-3 | *STEAP3* | cg25713625 | 120022835 | 3'UTR | S_Shore | 0.02713 |
| GOS-12 | *LRP1* | cg19559587 | 57588144 | Body;TSS200 | OpenSea | 0.02728 |
| GOS-12 | *TFRC* | cg21494636 | 195808986 | 1stExon;5'UTR;TSS200 | Island | 0.02732 |
| CV | *SLC11A1* | cg05190002 | 219262192 |  | N_Shore | 0.02744 |
| GOS-3 | *LRP1* | cg19559587 | 57588144 | Body;TSS200 | OpenSea | 0.02774 |
| GOS-12 | *FTL* | cg05617973 | 49468917 | Body | Island | 0.02790 |
| Death-3 | *CALR* | cg27180563 | 13056485 | TSS200 | Island | 0.02849 |
| Death-3 | *HMOX2* | cg05856951 | 4545328 | TSS1500;5'UTR | OpenSea | 0.03185 |
| GOS-3 | *PCBP1* | cg19638749 | 70312615 |  | N_Shore | 0.03195 |
| Death-12 | *LRP1* | cg22339313 | 57578642 | Body | N_Shore | 0.03249 |
| Death-12 | *SLC46A1* | cg00497630 | 26733052 | 1stExon | Island | 0.03250 |
| GOS-12 | *TFR2* | cg19767562 | 100224437 | Body | Island | 0.03330 |
| Death-12 | *TNF* | cg24540603 | 31547917 |  | N_Shore | 0.03351 |
| GOS-12 | *STEAP3* | cg25508118 | 119981769 | 5'UTR | Island | 0.03382 |
| Death-3 | *SLC11A1* | cg07719512 | 219246576 | TSS200 | OpenSea | 0.03473 |
| Death-3 | *STEAP3* | cg06872331 | 119981960 | 5'UTR | S_Shore | 0.03510 |
| CV | *TFR2* | cg02286663 | 100239307 | TSS200 | OpenSea | 0.03615 |
| Death-12 | *TNF* | cg21467614 | 31543638 | 1stExon | OpenSea | 0.03642 |
| DCI | *TNF* | cg21370522 | 31543219 | TSS200 | OpenSea | 0.03729 |
| GOS-12 | *TFRC* | cg09470983 | 195805948 | 5'UTR | N_Shelf | 0.03842 |
| DCI | *GSTP1* | cg11566244 | 67351786 | Body | Island | 0.03910 |
| Death-12 | *TNF* | cg15989608 | 31545321 | 3'UTR | N_Shelf | 0.03961 |
| Death-12 | *TFR2* | cg19767562 | 100224437 | Body | Island | 0.04019 |
| DCI | *SLC25A37* | cg01770362 | 23385913 | TSS1500 | N_Shore | 0.04043 |
| GOS-12 | *APP* | cg23269692 | 27372446 | Body | OpenSea | 0.04106 |
| GOS-12 | *LRP1* | cg22339313 | 57578642 | Body | N_Shore | 0.04190 |
| Death-12 | *LRP1* | cg25260176 | 57569940 | Body | Island | 0.04206 |
| Death-3 | *TNF* | cg15989608 | 31545321 | 3'UTR | N_Shelf | 0.04290 |
| DCI | *STEAP3* | cg25101327 | 119981142 | TSS1500 | Island | 0.04363 |
| Death-12 | *GSTP1* | cg11566244 | 67351786 | Body | Island | 0.04369 |
| Death-3 | *CALR* | cg06907141 | 13057206 | Body | S_Shore | 0.04432 |
| Death-12 | *LRP1* | cg01276169 | 57569898 | Body | Island | 0.04484 |
| GOS-12 | *GLRX5* | cg23581793 | 95999775 | Body | N_Shore | 0.04709 |
| CV | *TNF* | cg21370522 | 31543219 | TSS200 | OpenSea | 0.04821 |
| Death-3 | *STEAP3* | cg12535642 | 120020901 | Body | Island | 0.04823 |
| DCI | *APP* | cg18274664 | 27372461 | Body | OpenSea | 0.04857 |
| GOS-12 | *LRP1* | cg09749862 | 57584720 | Body | OpenSea | 0.04863 |
| DCI | *HFE2* | cg15553912 | 145415659 | 5'UTR;Body | Island | 0.04966 |
| Death-12 | *TNF* | cg02581828 | 31546769 |  | N_Shore | 0.04977 |

Binary logistic regression associations of CTH-adjusted trajectory group with patient outcomes while controlling for age, sex, self-identified race, and Fisher grade; associations with p-values <0.05 presented here; table sorted by p-value; CTH, cell type heterogenetiy; CV, Cerebral vasospasm; DCI, Delayed cerebral ischemia; GOS-3, Glasgow Outcome Scale at 3 months; GOS-12, Glasgow Outcome Scale at 12 months; Death-3, death at 3 months; Death-12, death at 12 months; ^a^UCSC Genome Browser Build GRCh37/hg19; ^b^Trimmed to unique listings, multiple listings indicate information for multiple transcripts; TSS, transcription start site; TSS200 = 0-200 bases upstream of the TSS; TSS1500 = 200-1500 bases upstream of the TSS; 5'UTR = 5' untranslated region, between the TSS and the ATG start site; Body = Between the ATG and stop codon; irrespective of the presence of introns, exons, TSS, or promoters; 3'UTR = between the stop codon and poly A tail; ^c^Shore, 0-2 kb from island; Shelf, 2-4 kb from island; N, upstream (5') of CpG island; S, downstream (3') of CpG island

**Figure S1.** Flow chart for prioritization of findings for replication

Budgetary constraints allowed only for replication of our top three hits. Following gene-specific data screening and analysis, top hits for replication were prioritized based on the strength of the identified associations, consistency of results after adjustment for CTH, and presence of hotspots (i.e., multiple CpG sites near each other associated with outcomes)

Table S5. Summary of prioritization of top hits in base models (i.e., unadjusted for CTH to allow for replication by MethySeq)

| Candidate gene | CpG Site(s) | Notes | Direction of Effect |
| --- | --- | --- | --- |
| *APP* | cg08866780 | Smallest p-value overall; significantly associated with GOS at 12 months and death at 3 and 12 months; suggestively associated with CV and GOS at 3 months; findings persistent after correction for CTH | Three groups (low, intermediate, and high DNA methylation); Increased DNA methylation associated with worse outcomes |
| *TNF* | cg12681001; cg21222743; cg10717214; cg21467614; **cg08553327^a^**; cg26729380; cg10650821  ^a^Chosen for replication because CpG site had the smallest p-values within the *TNF* gene | Second smallest p-value overall; located in a ‘hotspot’ of CpGs significant associated with GOS at 3 months and suggestively associated with GOS at 12 months and death at 3 and 12 months; GBTA model at cg08553327 did not pass post-GBTA diagnostics after correction for CTH so unable to compare findings with CTH corrected model, however, two CpG sites within the ‘hotspot’ did pass post-GBTA diagnostics and findings with GOS at 3 months were persistent after correction for CTH | Three groups (low, intermediate, and high DNA methylation); Intermediate DNA methylation associated with better outcomes compared with both low and high DNA methylation |
| *STEAP3* | cg25713625 | Third smallest p-value overall; significantly associated with GOS at 3 and 12 months and suggestively associated with death at 3 and 12 months; after correction for CTH, GOS findings were persistent | Three groups (low, intermediate, and high DNA methylation); Increased DNA methylation associated with worse outcomes |
| *SLC25A37* | cg18186478 | Significantly associated with GOS at 3 months and suggestively associated with GOS at 12 months and death at 3 and 12 months; GBTA model did not pass post-GBTA diagnostics after correction for CTH so unable to compare findings with CTH corrected model | Two groups (low and high DNA methylation); Increased DNA methylation associated with better outcomes |
| *ACO1* | cg13977526 | Significantly associated with GOS and death at 12 months; suggestively associated with death at 3 months; GBTA model did not pass post-GBTA diagnostics after correction for CTH so unable to compare findings with CTH corrected model | Two groups (low and high DNA methylation); Increased DNA methylation associated with worse outcomes |
| *FXN* | cg02268013; cg21229044 | Significantly associated with GOS at 3 months and suggestively associated with GOS at 12 months and death at 3 and 12 months; for cg21229044 GBTA controlling for CTH did pass post-GBTA diagnostics, but variability washed out resulting in one group only; GBTA model did not pass post-GBTA diagnostics after correction for CTH for cg02268013 so unable to compare findings with CTH corrected model; odds ratios observed between the two CpG sites are in opposite directions with respect to the effect of DNA methylation | cg02268013: Two groups (low and high DNA methylation); Increased DNA methylation associated with worse outcomes  cg21229044: Three groups (low, intermediate, and high DNA methylation); Increased DNA methylation associated with better outcomes |
| *SLC11A2* | cg22826226 | Significantly associated with GOS at 3 months; suggestively associated with GOS at 12 months and death at 3 and 12 months; GBTA model did not pass post-GBTA diagnostics after correction for CTH so unable to compare findings with CTH corrected model | Two groups (low and high DNA methylation); Increased DNA methylation associated with better outcomes |
| *ACO2* | cg25636833 | Significantly associated with death at 3 months; suggestively associated with GOS at 3 and 12 months and death at 12 months; findings persistent after correction for CTH | Three groups (low, intermediate, and high DNA methylation); Increased DNA methylation associated with worse outcomes |

Table sorted by smallest p-value; cg08866780 (*APP*), cg08553327 (*TNF*), and cg25713625 (*STEAP3*) prioritized as “top hits” for replication

**Figure S2.** Discovery phase spaghetti plots depicting DNA methylation over 13 days post-aSAH for top hits in *STEAP3*, *APP*, and *TNF*

Spahgetti plots of discovery phase DNA methylation over 13 days post-aSAH colored by inferred trajectory group (corresponding to Figure 2) for (A) cg25713625 (*STEAP3*), (B) cg08866780 (*APP*), and (C) cg08553327 (*TNF*); Marginal histogram represent the number of participants with DNA methylation observations on days 1 (n=62), 2 (n=89), 3 (n=52), 4 (n=86), 5 (n=103), 6 (n=52), 7 (n=80), 8 (n=84), 9 (n=33), 10 (n=69), 11 (n=51), 12 (n=32), 13 (n=46), 14 (n=0); group-based trajectory analysis was performed in SAS using M values and converted to beta values for the plots depicted here

**Table S6.** Discovery and replication levels of DNA methylation as measured by beta values (corresponds with Figure 2)

|  | Discovery | | | | | | Replication | | |
| --- | --- | --- | --- | --- | --- | --- | --- | --- | --- |
|  | cg25713625 (*STEAP3*) | | | cg25713625 (*STEAP3*) + CTH | | | cg25713625 (*STEAP3*) | | |
|  | Day 1 | Day 7 | Day 13 | Day 1 | Day 7 | Day 13 | Day 1 | Day 7 | Day 13 |
| Group 3 (High) | 26.75% | 27.16% | 27.04% | 26.39% | 26.53% | 26.72% | 41.18% | 37.04% | 45.25% |
| Group 2 (Intermediate) | 24.48% | 23.32% | 23.81% | 24.34% | 23.48% | 23.92% | 34.17% | 33.73% | 32.26% |
| Group 1 (Low) | 21.24% | 20.10% | 20.50% | 21.82% | 20.40% | 21.09% | 15.87% | 29.28% | 21.32%^a^ |
|  | cg08866780 (*APP*) | | | cg08866780 (*APP*) + CTH | | | *APP*, Site 2 | | |
|  | Day 1 | Day 7 | Day 13 | Day 1 | Day 7 | Day 13 | Day 1 | Day 7 | Day 13 |
| Group 3 (High) | 4.70% | 4.33% | 3.98% | 4.72% | 4.38% | 3.96% | 4.33% | 3.80% | 3.19% |
| Group 2 (Intermediate) | 3.16% | 3.01% | 2.92% | 3.22% | 3.02% | 2.91% | NA | | |
| Group 1 (Low) | 2.28% | 2.05% | 2.17% | 2.31% | 2.08% | 2.15% | 4.12% | 7.50% | 10.39% |
|  | cg08553327 (*TNF*) | | | cg08553327 (*TNF*) + CTH | | | *TNF*, Site 3 | | |
|  | Day 1 | Day 7 | Day 13 | Day 1 | Day 7 | Day 13 | Day 1 | Day 7 | Day 13 |
| Group 3 (High) | 7.44% | 20.81% | 4.69% | NA^e^ | | | 22.92% | 24.22%^b^ | 0.21%^c^ |
| Group 2 (Intermediate) | 14.58% | 8.86% | 8.37% |  |  |  | 16.55% | 14.53% | 12.51% |
| Group 1 (Low) | 11.56% | 5.13% | 6.88% |  |  |  | 7.28%^d^ | 4.21% | 3.72% |

Table corresponds with Figure 2 and presents DNA methylation levels as measured by beta values; ^a^measurement from day 11; ^b^measurement from day 8; ^c^measurement from day 14; ^d^measurement from day 2; ^e^the CTH-adjusted trajectory model for cg08553327 (*TNF*) did not pass posterior model quality control and therefore is not included here

Table S7. Discovery phase participant characteristics by trajectory group for cg25713625 (*STEAP3*), cg08866780 (*APP*), and cg08553327 (*TNF*)

|  |  | Unadjusted for CTH | | | | Adjusted for CTH | | | |
| --- | --- | --- | --- | --- | --- | --- | --- | --- | --- |
| CpG Site | Variable | Group 1 (Low) | Group 2 (Intermediate) | Group 3 (High) | *p* | Group 1 (Low) | Group 2 (Intermediate) | Group 3 (High) | *p* |
| cg25713625 (*STEAP3*) | Group membership, n (%) | 29 (11.1) | 166 (63.8) | 65 (25.0) |  | 32 (12.3) | 160 (61.5) | 68 (26.2) |  |
|  | Age, mean (SD) | 52.3 (12.2) | 53.0 (10.8) | 53.6 (11.2) | 0.862^a^ | 52.3 (13.6) | 53.0 (10.6) | 53.6 (10.7) | 0.852^a^ |
|  | Sex, female, n (%) | 13 (44.8) | 118 (71.1) | 48 (73.8) | **0.011**^b^ | 15 (46.9) | 116 (72.5) | 48 (70.6) | **0.016**^b^ |
|  | Race, white, n (%) | 20 (69.0) | 147 (88.6) | 58 (89.2) | **0.013**^b^ | 23 (71.9) | 140 (87.5) | 62 (91.2) | **0.026**^b^ |
|  | Fisher Grade |  |  |  |  |  |  |  |  |
|  | *2* | 6 (20.7) | 54 (32.5) | 18 (27.7) | 0.558^b^ | 10 (31.3) | 45 (28.1) | 23 (33.8) | 0.659^b^ |
|  | *3* | 14 (48.3) | 80 (48.2) | 32 (49.2) |  | 13 (40.6) | 83 (51.9) | 30 (44.1) |  |
|  | *4* | 9 (31.0) | 32 (19.3) | 15 (23.1) |  | 9 (28.1) | 32 (20.0) | 15 (22.1) |  |
|  | Intervention, Coil, n (%) | 16 (55.2) | 99 (59.6) | 40 (61.5) | 0.845^b^ | 18 (56.3) | 97 (60.6) | 40 (58.8) | 0.889^b^ |
| cg08866780 (*APP*) | Group membership, n (%) | 63 (24.2) | 139 (53.5) | 58 (22.3) |  | 67 (25.8) | 140 (53.8) | 53 (20.4) |  |
|  | Age, Mean (SD) | 52.7 (10.5) | 52.5 (11.0) | 54.8 (11.6) | 0.393^a^ | 52.0 (10.7) | 52.4 (11.2) | 56.3 (10.7) | 0.058^a^ |
|  | Sex, Female, n (%) | 45 (71.4) | 91 (65.5) | 43 (74.1) | 0.429^b^ | 46 (68.7) | 95 (67.9) | 38 (71.7) | 0.875^b^ |
|  | Race, white, n (%) | 55 (87.3) | 120 (86.3) | 50 (86.2) | 0.979^b^ | 59 (88.1) | 122 (87.1) | 44 (83.0) | 0.691^b^ |
|  | Fisher Grade |  |  |  |  |  |  |  |  |
|  | *2* | 20 (31.7) | 43 (30.9) | 15 (25.9) | 0.887^b^ | 21 (31.3) | 44 (31.4) | 13 (24.5) | 0.879^b^ |
|  | *3* | 28 (44.4) | 67 (48.2) | 31 (53.4) |  | 31 (46.3) | 68 (48.6) | 27 (50.9) |  |
|  | *4* | 15 (23.8) | 29 (20.9) | 12 (20.7) |  | 15 (22.4) | 28 (20.0) | 13 (24.5) |  |
|  | Intervention, Coil, n (%) | 46 (73.0) | 76 (54.7) | 33 (56.9) | **0.043**^b^ | 45 (67.2) | 77 (55.0) | 33 (62.3) | 0.225^b^ |
| cg08553327 (*TNF*) | Group membership, n (%) | 175 (67.3) | 70 (26.9) | 15 (5.8) |  | NA | | | |
|  | Age, mean (SD) | 53.9 (11.1) | 51.1 (10.5) | 52.9 (11.9) | 0.199^a^ |  |  |  |  |
|  | Sex, female, n (%) | 119 (68.0) | 48 (68.6) | 12 (80.0) | 0.628^b^ |  |  |  |  |
|  | Race, White, n (%) | 152 (86.9) | 59 (84.3) | 14 (93.3) | 0.633^b^ |  |  |  |  |
|  | Fisher Grade |  |  |  |  |  |  |  |  |
|  | *2* | 51 (29.1) | 21 (30.0) | 6 (40.0) | 0.92^b^ |  |  |  |  |
|  | *3* | 87 (49.7) | 33 (47.1) | 6 (40.0) |  |  |  |  |  |
|  | *4* | 37 (21.1) | 16 (22.9) | 3 (20.0) |  |  |  |  |  |
|  | Intervention, Coil, n (%) | 114 (65.1) | 34 (48.6) | 7 (46.7) | **0.033**^b^ |  |  |  |  |

Participant characteristics by trajectory group for our top three hits; SD, standard deviation; Race, self-identified; ^a^One-way analysis of variance; ^b^Chi-square test

**Table S8.** Discovery phase patient outcome distributions by base model trajectory groups for cg25713625 (*STEAP3*), cg08866780 (*APP*), and cg08553327 (*TNF*)

|  | cg25713625 (*STEAP3*) | | | cg08866780 (*APP*) | | | cg08553327 (*TNF*) | | |
| --- | --- | --- | --- | --- | --- | --- | --- | --- | --- |
|  | Group 1 (Low) | Group 2 (Intermediate) | Group 3 (High) | Group 1 (Low) | Group 2 (Intermediate) | Group 3 (High) | Group 1 (Low) | Group 2 (Intermediate) | Group 3 (High) |
|  | n=29 (11.2) | n=166 (63.8) | n=65 (25.0) | n=63 (24.2) | n=139 (53.5) | n=58 (22.3) | n=175 (67.3) | n=70 (26.9) | n=15 (5.8) |
| CV, n (%) |  |  |  |  |  |  |  |  |  |
| *Yes* | 10 (34.5) | 54 (32.5) | 27 (41.5) | 29 (69.0) | 43 (30.9) | 19 (32.8) | 59 (33.7) | 26 (37.1) | 6 (40.0) |
| *No* | 8 (27.6) | 52 (31.3) | 17 (26.2) | 13 (20.6) | 41 (29.5) | 23 (39.7) | 48 (27.4) | 25 (35.7) | 4 (26.7) |
| *Unknown* | 11 (37.9) | 60 (36.1) | 21 (32.3) | 21 (33.3) | 55 (39.6) | 16 (27.6) | 68 (38.9) | 19 (27.1) | 5 (33.3) |
| DCI, n (%) |  |  |  |  |  |  |  |  |  |
| *Yes* | 14 (48.3) | 79 (47.6) | 34 (52.3) | 32 (50.8) | 64 (46.0) | 31 (53.4) | 85 (48.6) | 31 (44.3) | 11 (73.3) |
| *No* | 15 (51.7) | 86 (51.8) | 30 (46.2) | 30 (47.6) | 75 (54.0) | 26 (44.8) | 90 (51.4) | 37 (52.9) | 4 (26.7) |
| *Unknown* | 0 | 1 (0.6) | 1 (1.5) | 1 (1.6) | 0 | 1 (1.7) | 0 | 2 (2.9) | 0 |
| GOS-3, n (%) |  |  |  |  |  |  |  |  |  |
| *Unfavorable* | 4 (13.8) | 38 (22.9) | 29 (44.6) | 25 (39.7) | 27 (19.4) | 19 (32.8) | 34 (19.4) | 28 (40.0) | 9 (60.0) |
| *Favorable* | 19 (65.5) | 101 (60.8) | 23 (35.4) | 30 (47.6) | 86 (61.9) | 27 (46.6) | 106 (60.6) | 33 (47.1) | 4 (26.7) |
| *Unknown* | 6 (20.7) | 27 (16.3) | 13 (20.0) | 8 (12.7) | 26 (18.7) | 12 (20.7) | 35 (20.0) | 9 (12.9) | 2 (13.3) |
| GOS-12, n (%) |  |  |  |  |  |  |  |  |  |
| *Unfavorable* | 2 (6.9) | 29 (17.5) | 22 (33.8) | 21 (33.3) | 14 (10.1) | 18 (31.0) | 29 (16.6) | 18 (25.7) | 6 (40.0) |
| *Favorable* | 22 (75.9) | 97 (58.4) | 32 (49.2) | 29 (46.0) | 96 (69.1) | 26 (44.8) | 107 (61.1) | 37 (52.9) | 7 (46.7) |
| *Unknown* | 5 (17.2) | 40 (24.1) | 11 (16.9) | 13 (20.6) | 29 (20.9) | 14 (24.1) | 39 (22.3) | 15 (21.4) | 2 (13.3) |
| Death-3, n (%) |  |  |  |  |  |  |  |  |  |
| *Yes* | 1 (3.4) | 21 (12.7) | 17 (26.2) | 15 (23.8) | 9 (6.5) | 15 (25.9) | 19 (10.9) | 15 (21.4) | 5 (33.3) |
| *No* | 23 (79.3) | 130 (78.3) | 40 (61.5) | 42 (66.7) | 116 (83.5) | 35 (60.3) | 135 (77.1) | 49 (70.0) | 9 (60.0) |
| *Unknown* | 5 (17.2) | 15 (9.0) | 8 (12.3) | 6 (9.5) | 14 (10.1) | 8 (13.8) | 21 (12.0) | 6 (8.6) | 1 (6.7) |
| Death-12, n (%) |  |  |  |  |  |  |  |  |  |
| *Yes* | 2 (6.9) | 23 (13.9) | 19 (29.2) | 17 (27.0) | 11 (7.9) | 16 (27.6) | 22 (12.6) | 16 (22.9) | 6 (40.0) |
| *No* | 22 (75.9) | 103 (62.0) | 35 (53.8) | 33 (52.4) | 99 (71.2) | 28 (48.3) | 114 (65.1) | 39 (55.7) | 7 (46.7) |
| *Unknown* | 5 (17.2) | 15 (9.0) | 8 (12.3) | 6 (9.5) | 14 (10.1) | 8 (13.8) | 21 (12.0) | 6 (8.6) | 1 (6.7) |

Base models, unadjusted for cell-type heterogeneity; CV, Cerebral Vasospasm; DCI, Delayed Cerebral Ischemia; GOS-3, Glasgow Outcome Scale at 3-months (Unfavorable=1-3; Favorable=4-5); GOS-12, Glasgow Outcome Scale at 12-months (Unfavorable=1-3; Favorable=4-5); Death-3, death at 3-months; Death-12, death at 12-months

**Figure S3.** Sankey plots depicting shifts in trajectory group assignment between discovery phase base and CTH-adjusted models

**
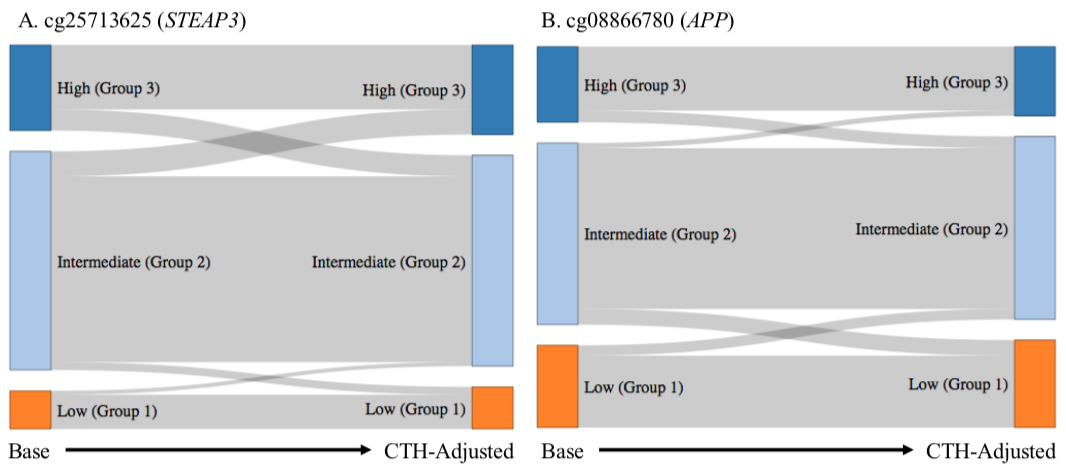
**

CTH, cell-type heterogeneity; Sankey plots depicting shifts in trajectory group assignment between base models (not adjusted for CTH, shown on the left side of each plot) and CTH-adjusted models (shown on the right side of each plot) for (A) cg25713625 (*STEAP3*) and (B) cg08866780 (*APP*); CTH-adjusted model for TNF did not pass quality control so could not be included here

**Table S9.** Results of discovery phase binary logistic regression examining associations of continuous cg25713625 (*STEAP3*) CSF DNA methylation with patient outcomes while controlling for age, sex, self-identified race, and Fisher grade in cross-sectional time points

|  |  | Time 1 (Days 1 to 2), n=157 | | | Time 2 (Days 3 to 5), n=242 | | | |
| --- | --- | --- | --- | --- | --- | --- | --- | --- |
| Outcome | n | OR | 95% CI | *p* | n | OR | 95% CI | *p* |
| GOS-3 | 129 | 4.17 | 0.65 to 29.83 | 0.141 | 196 | 9.69 | 2.26 to 46.66 | **0.003** |
| GOS-12 | 120 | 3.35 | 0.44 to 28.71 | 0.253 | 185 | 11.37 | 2.42 to 60.67 | **0.003** |
| Death-3 | 138 | 4.82 | 0.49 to 54.25 | 0.186 | 209 | 11.90 | 2.35 to 67.36 | **0.004** |
| Death-12 | 120 | 10.40 | 1.09 to 122.39 | **0.0495** | 185 | 10.27 | 2.08 to 57.35 | **0.006** |
|  | Time 3 (Days 6 to 8), n=214 | | | | Time 4 (Days 9 to 11), n=148 | | | |
| Outcome | n | OR | 95% CI | *p* | n | OR | 95% CI | *p* |
| GOS-3 | 169 | 10.61 | 2.22 to 57.02 | **0.004** | 116 | 45.83 | 6.70 to 422.65 | **0.0003** |
| GOS-12 | 162 | 10.49 | 2.08 to 59.61 | **0.006** | 112 | 20.35 | 2.87 to 188.21 | **0.004** |
| Death-3 | 185 | 4.98 | 0.88 to 30.39 | 0.074 | 128 | 8.22 | 1.08 to 86.88 | **0.008** |
| Death-12 | 162 | 9.04 | 1.65 to 55.54 | **0.014** | 112 | 8.94 | 1.14 to 87.56 | **0.046** |
|  | Time 5 (Days 12 to 13), n=94 | | | |  |  |  |  |
| Outcome | n | OR | 95% CI | *p* |  |  |  |  |
| GOS-3 | 70 | 5.54 | 0.62 to 58.27 | 0.133 |  |  |  |  |
| GOS-12 | 66 | 3.56 | 0.35 to 38.03 | 0.277 |  |  |  |  |
| Death-3 | 80 | 0.84 | 0.03 to 19.35 | 0.912 |  |  |  |  |
| Death-12 | 66 | 0.77 | 0.04 to 11.42 | 0.851 |  |  |  |  |

Phenotype ~ cg25713625_CSF + age + sex + fisher_grade + race; DNAm data analyzed as M values

**Table S10.** Characteristics of the subset of participants with blood DNA methylation available on days 1-2 post-aSAH

| Variable | Discovery sample (n=260) | | Subset of discovery sample with CSF and blood DNA methylation on days 1-2 post-aSAH (n=67) | |
| --- | --- | --- | --- | --- |
| Age, mean years (SD) | 53.1 (11.0) | | 52.8 (11.2) | |
| Sex, female, n (%) | 179 (68.8) | | 45 (67.2) | |
| Self-identified race, white, n (%) | 225 (86.5) | | 57 (85.1) | |
| Treatment, coil embolization, n (%) | 159 (61.2) | | 43 (64.2) | |
| Fisher grade, n (%) |  | |  | |
| 2 | 78 (30.0) | | 17 (25.4) | |
| 3 | 126 (48.5) | | 36 (53.7) | |
| 4 | 56 (21.5) | | 14 (20.9) | |
| Outcome | n | Unfavorable, n (%) | n | Unfavorable, n (%) |
| GOS-3 | 214 | 71 (33.2) | 52 | 20 (38.5) |
| GOS-12 | 204 | 53 (26.0) | 48 | 10 (20.8) |
| Death-3 | 232 | 39 (16.8) | 58 | 6 (10.3) |
| Death-12 | 204 | 44 (21.6) | 48 | 8 (16.7) |

aSAH, aneurysmal subarachnoid hemorrhage; SD, standard deviation; CV, cerebral vasospasm (unfavorable=CV present); DCI, delayed cerebral ischemia (unfavorable=DCI present); GOS-3, Glasgow Outcome Scale at 3 months (unfavorable=1-3); GOS-12, Glasgow Outcome Scale at 12 months (unfavorable=1-3); Death-3, death at 3 months (unfavorable=yes); Death-12, death at 12 months (unfavorable=yes)

**Figure S4.** Correlation between cg25713625 (*STEAP3*) CSF and Blood DNA Methylation, Days 1-2 post-aSAH


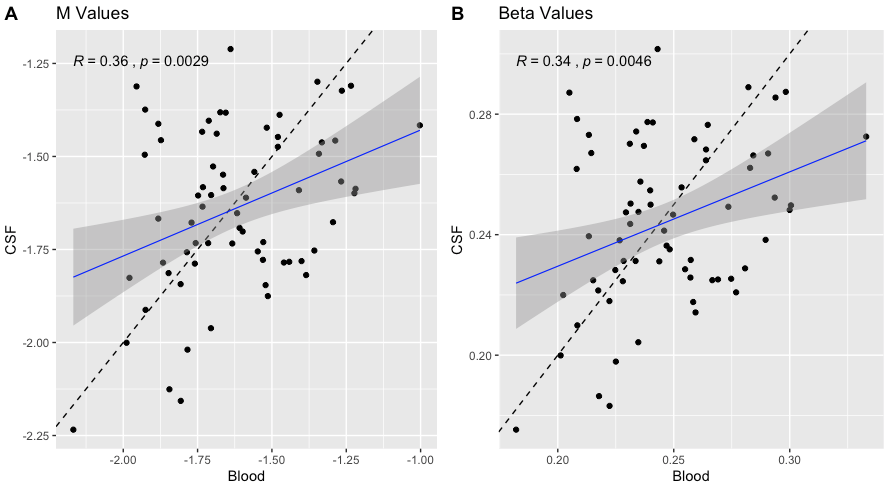


R, Pearson correlation coefficient; black dashed line, *y=x*; blue solid line, regression line fitted to the data

**Table S11**. Results of binary logistic regression examining associations of continuous cg25713625 (*STEAP3*) DNA methylation with patient outcomes while controlling for age, sex, self-identified race, and Fisher grade in the subset of participants with both blood and CSF available on days 1-2 post-aSAH in the discovery sample

|  |  | CSF^a^ | | | Blood^b^ | | |
| --- | --- | --- | --- | --- | --- | --- | --- |
| Outcome | n | OR | 95% CI | *p*^c^ | OR | 95% CI | *p*^c^ |
| GOS-3 | 52 | 7.830 | 0.36 to 272.45 | 0.215 | 0.137 | 0.007 to 2.00 | 0.162 |
| GOS-12 | 48 | 1.230 | 0.08 to 49.6 | 0.908 | 0.741 | 0.03 to 14.97 | 0.847 |
| Death-3 | 58 | 1.861 | 0.248 to 215.97 | 0.783 | 0.735 | 0.02 to 30.21 | 0.869 |
| Death-12 | 48 | 13.023 | 0.235 to 122.24 | 0.245 | 1.476 | 0.08 to 39.81 | 0.815 |

^a^Phenotype ~ cg25713625_CSF + age + sex + fisher_grade + race; ^b^Phenotype ~ cg25713625_blood + age + sex + fisher_grade + race; total n=67 (see Table S9 above); DNAm data analyzed as M values

**Figure S5.** Replication DNA methylation trajectory plots for CpGs near cg08866780 (*APP*)

Replication phase inferred trajectory groups computed using group-based trajectory analysis; group membership depicted as percent membership; bars plot the 95% confidence interval for the estimate; site positions/details provided below in Table S14; group-based trajectory analysis was performed in SAS using M values and converted to beta values for the plots depicted here

**Figure S6.** Replication DNA methylation trajectory plots for CpGs near cg08553327 (*TNF*)

Replication phase inferred trajectory groups computed using group-based trajectory analysis; group membership depicted as percent membership; bars plot the 95% confidence interval for the estimate; site positions/details provided below in Table S14; group-based trajectory analysis was performed in SAS using M values and converted to beta values for the plots depicted here

**Table S12.** Post-GBTA diagnostic summary for replication data for sites in *STEAP3*, *APP*, and *TNF*

| *STEAP3* | | | | | | |
| --- | --- | --- | --- | --- | --- | --- |
| Site | Position | Group | $\pi$ | P* | AvePP | OCC |
|  |  | 3 (High) | 26.20 | 26.00 | 0.84 | 14.66 |
| Site 1 of 1 (cg25713625) | chr2: 120022835 | 2 (Intermediate) | 67.51 | 68.00 | 0.93 | 6.354 |
|  |  | 1 (Low) | 6.29 | 6.00 | 0.98 | 807.8 |
| *APP* | | | | | | |
| Site | Position | Group | $\pi$ | P* | AvePP | OCC |
| Site 1 of 3 | chr21: 27543494 | 1 | NA | | | |
| Site 2 of 3 | chr21: 27543504 | 2 (High) | 91.20 | 92.93 | 0.97 | 2.95^a^ |
|  |  | 1 (Low) | 8.81 | 7.07 | 0.83 | 50.13 |
| Site 3 of 3 | chr21: 27543511 | 1 | NA | | | |
| *TNF* | | | | | | |
| Site | Position | Group | $\pi$ | P* | AvePP | OCC |
| Site 1 of 4 | chr6: 31543554 | 2 (High) | 2.83 | 2.02 | 0.99 | 4802.00 |
|  |  | 1 (Low) | 97.17 | 97.98 | 0.99 | 3.43^a^ |
| Site 2 of 4 | chr6: 31543561 | 2 (High) | 9.25 | 9.09 | 0.80 | 38.87 |
|  |  | 1 (Low) | 90.75 | 90.91 | 0.98 | 4.55^a^ |
| Site 3 of 4 | chr6: 31543569 | 3 (High) | 3.31 | 3.03 | 0.99 | 3523.00 |
|  |  | 2 (Intermediate) | 89.54 | 90.91 | 0.98 | 5.03 |
|  |  | 1 (Low) | 7.14 | 6.06 | 0.84 | 70.76 |
| Site 4 of 4 | chr6: 31543607 | 2 (High) | 6.19 | 6.06 | 0.84 | 78.40 |
|  |  | 1 (Low) | 93.81 | 93.94 | 0.99 | 5.51 |

Table corresponds with Figure 2c (*STEAP3*), Figure S5 (*APP*), and Figure S6 (*TNF*); replication data collected for *STEAP3* based on discovery phase association at cg25713625; replication data collected for *APP* based on discovery phase association at cg08866780; replication data collected for *TNF* based on discovery phase association at cg08553327; $\pi$, population size of the trajectory group, j (i.e., the probability that a randomly selected individual belongs to group j); P*, observed group proportion; AvePP, average posterior probability; OCC, odds of correct classification; ^a^fail OCC criteria <5; NA, not applicable because only one trajectory group was inferred from data; GBTA, group-based trajectory analysis

**Table S13.** Replication results of binary logistic regression examining associations of *TNF* sites 3 and 4 near cg08553327 (*TNF*), with patient outcomes while controlling for age, sex, self-identified race, and Fisher grade

| Site 3, Polynomial Order 022 | | | | | | |
| --- | --- | --- | --- | --- | --- | --- |
|  | Group 2 (Intermediate) vs. Group 1 (Low) | | | Group 2 (Intermediate) vs. Group 1 (Low) | | |
| Outcome | OR | 95% CI | *p* | OR | 95% CI | *p* |
| GOS-3 | 0.294 | 0.03 to 2.66 | 0.2842 | 2.320 | 0.06 to 113.07 | 0.6468 |
| GOS-12 | 0.231 | 0.02 to 2.09 | 0.1986 | 2.043 | 0.05 to 99.21 | 0.6957 |
| Death-3 | 0.137 | 0.01 to 1.10 | 0.0718 | 1.731 | 0.05 to 79.5 | 0.7593 |
| Death-12 | 0.191 | 0.02 to 1.83 | 0.1568 | 2.500 | 0.06 to 138.91 | 0.6292 |
| Site 4, Polynomial Order 10 | | | | | | |
|  | Group 2 (High) vs. Group 1 (Low) | | | NA | | |
| Outcome | OR | 95% CI | *p* |  |  |  |
| GOS-3 | 3.285 | 0.34 to 38.61 | 0.3064 |  |  |  |
| GOS-12 | 3.967 | 0.42 to 45.43 | 0.2299 |  |  |  |
| Death-3 | 6.381 | 0.78 to 70.34 | 0.0941 |  |  |  |
| Death-12 | 4.695 | 0.48 to 56.65 | 0.1878 |  |  |  |

Corresponds to trajectory groups depicted in Figure S7; GOS-3, Glasgow Outcome Scale at 3-months (Unfavorable=1-3); GOS-12, Glasgow Outcome Scale at 12-months (Unfavorable=1-3); Death-3, death at 3-months; Death-12, death at 12-months

**Figure S7.** Comparison of cg25713625 (*STEAP3*) DNA methylation data from validation samples overlapping between discovery and replication data


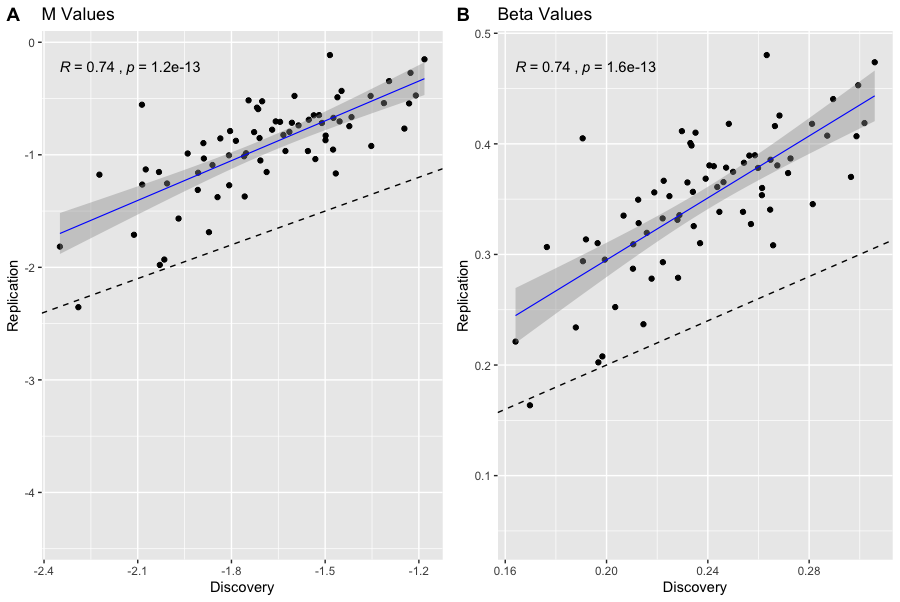


Comparison of DNA methylation data for 22 participants included in both the discovery and replication data collection (n=72 validation samples) using (A) M values and (B) Beta values; R, Pearson correlation coefficient; black dashed line, *y=x*; blue solid line, regression line fitted to the data

**Figure S8.** Sankey plot depicting shifts in trajectory group assignment between discovery and replication analyses for cg25713625 (*STEAP3*) replication


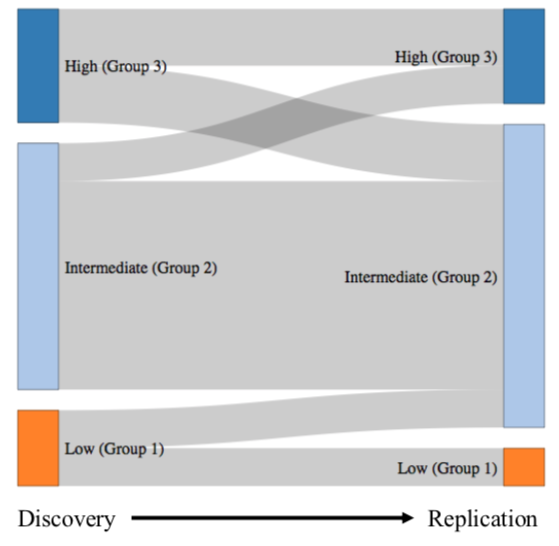


Sankey plots depicting shifts in trajectory group assignment between discovery phase (shown on the left side of the plot) and replication phase (shown on the right side of the plot); plot created using the 22 participants included in both the discovery and replication data collection (n=72 validation samples) and shows that 16 of the 22 validation participants remained in the same group from the discovery to replication phases

***Section II*: Expanded replication data collection methods**^2^

The replication sample was recruited together with the discovery sample. Longitudinal replication DNAm data were generated for our top hits using bisulfite methylation sequencing/pyrosequencing (i.e., MethylSeq) at the Center for Inherited Disease Research. Using 500 ng of DNA, bisulfite conversion was carried out with the Qiagen EpiTect Bisulfite kit (Catalog Number 59104 [48 reactions] or 59110 [96 reactions]) and PCR reactions were completed using the Qiagen Pyromark PCR Kit (Catalog number 978703 [200 reactions] or 978705 [800 reactions]) following standard protocols and assays designed by and proprietary to Qiagen (Table S2). Sequencing was completed on Qiagen Pyromark Q48 Autoprep instrument using recommended reagents (Catalog number 974002 and 974022) and following the instrument protocol (firmware v.4.03, software v.4.2.1, OS v.1.1.2). PCR amplification for CG08866780_93 bp (*APP*) did not amplify well, so samples were run using a standard low concentration protocol which implements PyroMark Q48 magnetic beads (Catalog number 974203). Data were called using the Pyromark Q48 Autoprep 2.4.2 software. Probe sequences were designed to capture top hits and regions surrounding top hits. For cg08866780 (*APP*) and cg08553327 (*TNF*), this captured the target CpG sites and additional variable CpG sites in this region for a total of 4 sites (*APP*) and 8 sites (*TNF*). As part of our QC filtering, sites with >5% of samples failing MethylSeq were excluded (Table S3). For our top hits of interest in *STEAP3*, *APP*, and *TNF*, we examined a total of 1, 3, and 4 replication sites, respectively. Samples with incomplete conversion were not used for sequencing and samples that failed the MethylSeq assay were excluded. A subset of the discovery sample (N=72 total samples for n=22 participants) were re-assayed by MethylSeq along with the replication samples. The total number of participants with MethylSeq data was N=122, while the number of independent (i.e., non-overlapping with the discovery sample) participants for final replication analysis was n=100.

**Table S14.** Replication assay information for top hits


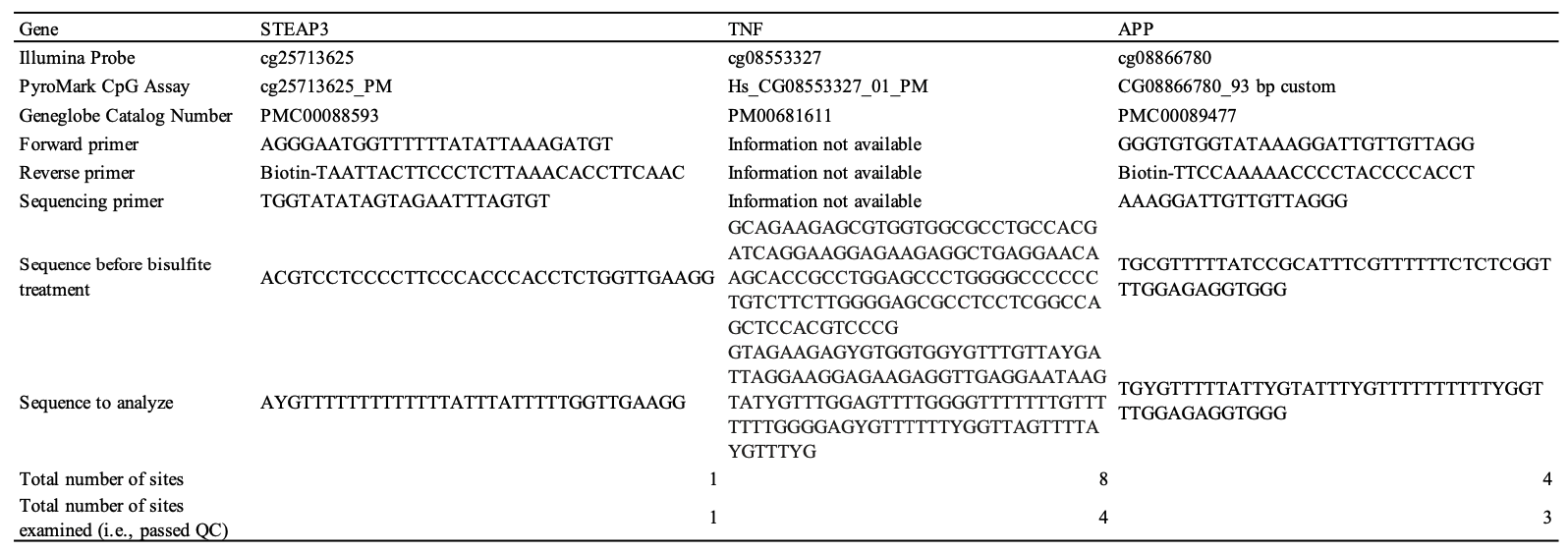


Note: Site positions provided in table below

**Table S15.** Summary of replication data QC (pass, check, and fail)

|  | Position^a^ | Pass | Check | Fail | NA | Total | % Fail |
| --- | --- | --- | --- | --- | --- | --- | --- |
| *STEAP3* | | | | | | | |
| Site 1 (cg25713625) | chr2: 120022835 | 370 | 5 | 2 | 2 | 379 | 0.53 |
| *APP* | | | | | | | |
| Site 1 | chr21: 27543494 | 369 | 6 | 0 | 0 | 375 | 0.00 |
| Site 2 | chr21: 27543504 | 368 | 7 | 0 | 0 | 375 | 0.00 |
| Site 3 | chr21: 27543511 | 356 | 17 | 2 | 0 | 375 | 0.53 |
| Site 4 (cg08866780) | chr21: 27543523 | 0 | 10 | 365 | 0 | 375 | 97.33* |
| *TNF* | | | | | | | |
| Site 1 | chr6: 31543540 | 368 | 7 | 2 | 3 | 380 | 0.53 |
| Site 2 | chr6: 31543545 | 369 | 6 | 2 | 3 | 380 | 0.53 |
| Site 3 | chr6: 31543557 | 369 | 6 | 2 | 3 | 380 | 0.53 |
| Site 4 | chr6: 31543565 | 346 | 28 | 3 | 3 | 380 | 0.80 |
| Site 5* | chr6: 31543603 | 67 | 281 | 29 | 3 | 380 | 7.69* |
| Site 6* | chr6: 31543638 | 0 | 141 | 236 | 3 | 380 | 62.60* |
| Site 7 (cg08553327)* | chr6: 31543647 | 60 | 288 | 29 | 3 | 380 | 7.69* |
| Site 8* | chr6: 31543655 | 60 | 288 | 29 | 3 | 380 | 7.69* |
| Replication data collected for *STEAP3* based on discovery phase association at cg25713625; replication data collected for *APP* based on discovery phase association at cg08866780; replication data collected for *TNF* based on discovery phase association at cg08553327; *Site excluded because >5% of samples failed; ^a^UCSC Genome Browser GRCh37/hg 19 | | | | | | | |

***Section III*: Expanded statistical analysis**

***Preliminary analyses***

Standard descriptive statistics were computed for all independent, dependent, and potentially confounding/covariate data given the variable’s level of measurement (e.g., means and standard deviations for continuous type normally distributed variables, frequency counts and percentages for categorical variables). Group comparisons were performed using t-tests to evaluate equality of means for continuous type variables and Pearson’s chi-square test of independence or Fisher’s exact test for equality of proportions for categorical variables. Preliminary analyses were conducted to identify potential confounders/covariates. Known predictors of outcomes after aSAH include degree of hemorrhage (as measured by the clinical grading scale, Fisher grade), age, race, and sex.^3^

Data screening procedures were performed given the variable’s level of measurement. Data were screened for accuracy using frequency tables for categorical variables (e.g., sex, race) and summary statistics (mean, standard deviation, minimum, and maximum) for continuous variables (e.g., age, DNA methylation). To identify outliers, data were examined in frequency tables for categorical variables and in histograms and sina plots for continuous variables. If potential outliers were detected, data were inspected for accuracy and the influence of data were evaluated in a sensitivity analysis. Because GBTA is intended to identify distinct groups with shared patterns, it is sensitive to outliers. For DNA methylation data, any DNA methylation value labeled as an extreme outlier (a DNA methylation value above or below three times the interquartile range) was replaced with the maximum or minimum observed DNA methylation value below the extreme outlier threshold for values on each day. The decision to modify extreme outliers in this way was made to meet our trajectory group size requirement of 5% (detailed below) and to retain as many CpG sites as possible in our patient outcome association testing, though we acknowledge this is a limitation of our study if the outlying samples truly belong to a distinct group. Data were screened for independence using scatterplots, multicollinearity using tolerance, variance inflation factors, and condition indices with variance decomposition proportions, and normality using histograms with a normal curve overlay and Kolmogorov-Smirnov test of normality. Missing data were assessed and filled in from the medical record when possible.

***Group-based trajectory analyses***

Group-based trajectory analysis (GBTA) for DNA methylation data was conducted in SAS using Proc TRAJ assuming a censored normal model, which helps correct for right censored data within longitudinal studies. DNA methylation data were analyzed as M values, and resulting trajectories were converted to beta values for presentations in plots in the main text and supplementary information. For individual CpG sites within our candidate gene DNA methylation data, the change pattern over time was examined to infer distinct trajectory patterns/groups of site-specific methylation across time over the acute recovery period post-aSAH. In GBTA, models with a varying number of groups and polynomial orders (group trajectory shapes) are compared to find the model that best fits the longitudinal data.^4^ As part of this modeling process, participants are assigned a posterior probability of assignment for each trajectory group in a model; each participant is then assigned to whichever group they have the highest probability of being in.^4^ Given the subjectivity required in traditional GBTA modeling and the large number of candidate genes and CpG sites analyzed in this study, the model selection process was largely automated for 39 possible models with a maximum of three groups and comprehensive combinations of polynomial orders of 0 (intercept only), 1 (linear), and 2 (quadratic).

Our GBTA automated protocol has been described in detail as part of our pilot work.^1^ Briefly, out of the 39 possible models, a best fitting ‘preliminary’ model was identified based on the maximum Bayesian Information Criterion (BIC). In general, BIC measures improvement in model fit gained with the estimation of more parameters such as an increased number of groups and/or more complex trajectory shapes, but also applies a penalty for model complexity. In SAS, a larger BIC value indicates a better model fit. Using GBTA in SAS, two BIC values were computed based on (1) the number of participants and (2) the total number of methylation observations over time. The true BIC falls somewhere within these values.^4^ In cases where the BICs did not agree on the best preliminary model, we assigned a ‘simplicity score’ to assist in identifying the more parsimonious model (e.g., 000 as a simpler model than 111), which was carried forward. Following selection of a preliminary model for each CpG site, we performed a secondary evaluation of model adequacy (i.e., post-GBTA diagnostics) using several traditional diagnostics including (1) an average posterior probability (AvePP) >0.7, (2) odds of correct classification (OCC) >5, (3) estimated group membership (π) >5%, (4) reasonably close estimated group membership (π) versus the assigned group proportion (P*), and (5) a relatively narrow 95% confidence interval for the estimated group probability (π).^5^ For CpG sites which preliminary models failed secondary evaluation, the ‘next best’ fitting model was selected and secondary evaluation was performed again. If preliminary models for a CpG site failed a second time, we concluded that DNA methylation trajectory groups could not be inferred with high accuracy for that site and it was excluded from further analysis.^1^

To examine the potential clinical utility of DNA methylation trajectories unadjusted for CTH as well as evaluate potential confounding by cell-type, we implemented our GBTA protocol twice for all CpG sites to compute DNA methylation trajectories both unadjusted for CTH and adjusted for CTH.^1^ As part of the data cleaning and QC pipeline, CTH data were generated using Houseman’s reference-free method.^6^ These data include percentages of five putative cell types for each biosample and were controlled for as time-varying covariates during GBTA in our CTH-adjusted models.

Trajectory group – patient outcome associations

Finally, binary logistic regression was performed in R to determine the relationship between inferred trajectory groups for each DNA methylation site and patient outcomes while controlling for age, sex, race, and Fisher grade. A likelihood ratio test was used to produce a global p-value of the overall model fit by comparing the full model (including the CpG site) with a restricted model (omitting the CpG site). Given the correlation between patient outcomes in this study, permutation testing, rather than strict Bonferroni corrections, was used to correct for testing correlated outcomes.^1^ This was done by shuffling the independent variables (i.e., trajectory groups) 10,000 times, performing binary logistic regression for each outcome, and recording the minimum p-value across the outcome-specific results. In the distribution of 10,000 simulated null minimum p-values, the value that occurred at the 95^th^ percentile was taken to be the empirical significance threshold T.

**References**

1. Heinsberg LW, Arockiaraj AI, Crago EA, Ren D, Shaffer JR, Sherwood PR, Sereika SM, Weeks DE, Conley YP. Genetic Variability and Trajectories of DNA Methylation May Support a Role for HAMP in Patient Outcomes After Aneurysmal Subarachnoid Hemorrhage. *Neurocrit Care*. 2020;32(2):550-563. doi:10.1007/s12028-019-00787-4

2. Heinsberg L. Multi-Omics of the Iron Homeostasis Pathway in Patient Outcomes after Aneurysmal Subarachnoid Hemorrhage [PhD Thesis]. Published online 2020. http://d-scholarship.pitt.edu/39224/

3. Jabbarli R, Reinhard M, Roelz R, Shah M, Niesen W-D, Kaier K, Taschner C, Weyerbrock A, Velthoven V Van. The predictors and clinical impact of intraventricular hemorrhage in patients with aneurysmal subarachnoid hemorrhage. *Int J Stroke*. 2016;11(1):68-76. doi:10.1177/1747493015607518

4. Jones BL, Nagin DS, Roeder K. A SAS procedure based on mixture models for estimating developmental trajectories. *Sociol Methods Res*. 2001;29(3):374-393. doi:10.1177/0049124101029003005

5. Nagin DS. *Group-Based Modeling of Development*. Harvard University Press; 2005.

6. Houseman EA, Molitor J, Marsit CJ. Reference-free cell mixture adjustments in analysis of DNA methylation data. *Bioinformatics*. 2014;30(10):1431-1439. doi:10.1093/bioinformatics/btu029
